# Supplementary material for: Quantitative Sensory Testing in Fibromyalgia Syndrome: A Scoping Review
Source: Biomedicines. 2025 Apr 17;13(4):988. doi: 10.3390/biomedicines13040988 (PMC12025226; doi:10.3390/biomedicines13040988)
Supplement: Supplementary file 1 [file biomedicines-13-00988-s001.zip › biomedicines-3547230-supplementary.pdf]

Supplementary Material

Supplementary S1. Preferred Reporting Items for Systematic reviews and Meta-Analyses extension for Scoping Reviews (PRISMA-ScR) Checklist

SECTION	ITEM	PRISMA-ScR CHECKLIST ITEM	REPORTED ON PAGE #
**TITLE**			
Title	1	Identify the report as a scoping review.	1
**ABSTRACT**			
Structured summary	2	Provide a structured summary that includes (as applicable) background, objectives, eligibility criteria, sources of evidence, charting methods, results, and conclusions that relate to the review questions and objectives.	2
**INTRODUCTION**			
Rationale	3	Describe the rationale for the review in the context of what is already known. Explain why the review questions/objectives lend themselves to a scoping review approach.	3
Objectives	4	Provide an explicit statement of the questions and objectives being addressed with reference to their key elements (e.g., population or participants, concepts, and context) or other relevant key elements used to conceptualize the review questions and/or objectives.	3
**METHODS**			
Protocol and registration	5	Indicate whether a review protocol exists; state if and where it can be accessed (e.g., a Web address); and if available, provide registration information, including the registration number.	3
Eligibility criteria	6	Specify characteristics of the sources of evidence used as eligibility criteria (e.g., years considered, language, and publication status), and provide a rationale.	4
Information sources*	7	Describe all information sources in the search (e.g., databases with dates of coverage and contact with authors to identify additional sources) as well as the date the most recent search was executed.	4, 5
Search	8	Present the full electronic search strategy for at least one database, including any limits used, such that it could be repeated.	4
Selection of sources of evidence†	9	State the process for selecting sources of evidence (i.e., screening and eligibility) included in the scoping review.	4
Data charting process‡	10	Describe the methods of charting data from the included sources of evidence (e.g., calibrated forms or forms that have been tested by the team before their use and whether data charting was performed independently or in duplicate) and any processes for obtaining and confirming data from investigators.	4, 5
Data items	11	List and define all variables for which data were sought and any assumptions and simplifications made.	5

SECTION	ITEM	PRISMA-ScR CHECKLIST ITEM	REPORTED ON PAGE #
Critical appraisal of individual sources of evidence§	12	If performed, provide a rationale for conducting a critical appraisal of included sources of evidence; describe the methods used and how this information was used in any data synthesis (if appropriate).	4
Synthesis of results	13	Describe the methods of handling and summarizing the data that were charted.	5
**RESULTS**			
Selection of sources of evidence	14	Provide the number of sources of evidence screened, assessed for eligibility, and included in the review, with reasons for exclusions at each stage, ideally using a flow diagram.	5, 6
Characteristics of sources of evidence	15	For each source of evidence, present characteristics for which data were charted and provide the citations.	6
Critical appraisal within sources of evidence	16	If performed, present data on critical appraisal of included sources of evidence (see item 12).	6
Results of individual sources of evidence	17	For each included source of evidence, present the relevant data that were charted that relate to the review questions and objectives.	6, 7
Synthesis of results	18	Summarize and/or present the charting results as they relate to the review questions and objectives.	6,7, 8
**DISCUSSION**			
Summary of evidence	19	Summarize the main results (including an overview of concepts, themes, and types of evidence available), link to the review questions and objectives, and consider the relevance to key groups.	8, 9
Limitations	20	Discuss the limitations of the scoping review process.	10
Conclusions	21	Provide a general interpretation of the results with respect to the review questions and objectives, as well as potential implications and/or next steps.	11
**FUNDING**			
Funding	22	Describe the sources of funding for the included sources of evidence as well as the sources of funding for the scoping review. Describe the role of the funders of the scoping review.	11

JBI = Joanna Briggs Institute; PRISMA-ScR = Preferred Reporting Items for Systematic reviews and Meta-Analyses extension for Scoping Reviews.
* Where *sources of evidence* (see second footnote) are compiled from, such as bibliographic databases, social media platforms, and Web sites.
† A more inclusive/heterogeneous term used to account for the different types of evidence or data sources (e.g., quantitative and/or qualitative research, expert opinion, and policy documents) that may be eligible in a scoping review as opposed to only studies. This is not to be confused with *information sources* (see first footnote).
‡ The frameworks by Arksey and O'Malley (6) and Levac and colleagues (7) and the JBI guidance (4, 5) refer to the process of data extraction in a scoping review as data charting.
§ The process of systematically examining research evidence to assess its validity, results, and relevance before using it to inform a decision. This term is used for items 12 and 19 instead of "risk of bias" (which is more applicable to systematic reviews of interventions) to include and acknowledge the various sources of evidence that may be used in a scoping review (e.g., quantitative and/or qualitative research, expert opinion, and policy document).

Checklist used to ensure adherence to the Preferred Reporting Items for Systematic Reviews and Meta-Analyses extension for Scoping Reviews (PRISMA-ScR). Each item indicates where it is addressed in the main manuscript.

From: Tricco AC, Lillie E, Zarin W, O'Brien KK, Colquhoun H, Levac D et al. PRISMA Extension for Scoping Reviews (PRISMAScR): Checklist and Explanation. Ann Intern Med. 2018;169:467–473. doi: 10.7326/M18-0850.

Supplementary S2: Search strategies for systematic review

Database	Search Date	Search Terms	Results (n)
Pubmed	June 03, 2024	((“fibromyalgia"[Title/Abstract] OR "fibromyalgia"[MeSH Terms]) AND ("quantitative sensory testing"[Title/Abstract] OR "QST"[Title/Abstract] OR "psychophysical test"[Title/Abstract] OR "allodynia"[Title/Abstract] OR "temporal summation"[Title/Abstract] OR "conditioned pain modulation"[Title/Abstract] OR "pressure pain threshold"[Title/Abstract]))	508
Embase	June 03, 2024	('fibromyalgia'/exp OR 'fibromyalgia':ti,ab) AND ('quantitative sensory testing':ti,ab OR 'QST':ti,ab OR 'psychophysical test*':ti,ab OR 'allodynia':ti,ab OR 'temporal summation':ti,ab OR 'conditioned pain modulation':ti,ab OR 'pressure pain threshold*':ti,ab)	817
Web of Science	June 03, 2024	TS=("fibromyalgia" AND ("quantitative sensory testing" OR "QST" OR "psychophysical test" OR "allodynia" OR "temporal summation" OR "conditioned pain modulation" OR "pressure pain threshold"))	1187

Notes:

Numbers (n), Terms restricted to abstract and title (ab:ti), title, abstract, and keywords (TITLE-ABS-KEY), expanded ('/exp), and Topic Search (TS).

Results were excluded if conference abstract/review, editorial, letter, note, or short survey.

Detailed description of the search strategies used across PubMed, Embase, and Web of Science, including search terms, filters applied, and the number of results retrieved from each database.

Supplementary S3: Rob2 from the RCT studies included (N=39)

Intention-to-treat	Unique ID	Study ID	Experimental	Comparator	Outcome	Weight	D1	D2	D3	D4	D5	Overall
	1	232	FMS+ active TENS (Transı	FMS+ placebo TENS / no T	Increase PPT	1	+	+	+	+	+	+
	2	41	FMS +Hyperbaric	FMS + Medication	Increase neuroplasticit	1	+	!	+	+	+	+
	3	195	FMS+ educational activity	FMS + control	Increase PPT	1	+	!	+	+	+	+
	4	45	FMS +multisensory stim.	FMS + placebo	pain response	1	+	+	+	+	+	+
	5	40	FMS + aquatic therapy (A	FMS + Land-based therapy	Improvement in pain	1	+	+	+	+	+	+
	6	12	FMS +radial shockwave tl	FMS + Placebo RSWT	Improvement PPT	1	+	+	+	+	+	+
	7	231	FMS +HBOT- Hyperbaric ı	FMS +medications	Improvement of FMS s	1	+	+	+	+	+	+
	8	32	FMS + isometric exercise	HC + isometric exercise	Improvement in CPM	1	+	+	+	+	+	+
	9	34	FMS + Transcutaneous n	FMS + sham	Pain reduction	1	+	+	+	+	+	+
	10	206	FM + Transcranial alterna	FMS + Sham HD-tACS	NA	1	+	+	+	+	+	+
	11	58	FMS + TMS (M1, DLPF, OI	FMS + TMS Sham	pain intensity	1	+	+	+	+	+	+
	12	64	FMS + Physical exercise	FMS + TMS /Control group	Improvement in pain	1	+	+	+	+	+	+
	13	67	FMS active wearable tran	FMS sham wearable trans	NA	1	+	+	+	+	+	+
	14	63	FMS + pregabalin and ex	FMS + exercise	NA	1	+	+	+	+	+	+
	15	71	FM +Qigong	FM+ Sham Qigong	Increase PPT	1	+	+	+	+	+	+
	16	70	FMS Neuroadaptive elect	FMS + Sham	Improvement in pain a	1	+	+	+	+	+	+
	17	86	FMS + low physical exerc	FMS +control	pain perception ; PPT	1	+	+	+	+	+	+
	18	17	FMS + pain neuroscience	NA	Improvement in pain s	1	+	+	+	+	+	+
	19	91	FMS + Tapaentadol	FMS + Placebo	Increase CPM	1	+	+	+	+	+	+
	20	49	FMS LDN/ or tDCS	FMS with placebo treamer	Improvement in functi	1	!	!	+	+	!	!
	21	30	FMS + milnacipran	FMS + placebo	Increase CPM	1	!	!	!	!	!	!
	22	123	FMS + general osteopath	NA	Pain intensity	1	+	+	+	+	+	+
	23	104	FMS + Exercise	FMS + control	Increase in VO2 and PF	1	+	+	+	+	+	+
	24	95	FMS + Electromyogram (I	FMS + control	Improvement PPT	1	+	+	+	+	+	+
	25	132	FMS + interdisciplinary gı	FMS + control group	Increase PPT and QoL	1	+	!	+	+	+	+
	26	145	FMS + HBOT	FMS control	Increase in PPT	1	+	+	+	+	+	+
	27	141	FMS +Milacipran	FMS +Placebo	Change in clinical pain/	1	+	!	+	+	!	!
	28	219	FMS +manual therapy	FMS + control	Improve PPT	1	+	+	+	+	+	+
	29	223	FMS + 100mg/200mg lidc	FMS + placebo	Reduce in clinical pain	1	+	+	+	+	+	+
	30	117	FMS + dry needling	FMS + control	Overall pain score	1	+	!	+	+	+	+
	31	29	FMS + pain physiology ed	FMS receiving self manage	Change on endogenou:	1	+	+	+	+	+	+
	32	171	FMS + tDCS	FMS + sham	Increase PPT	1	+	+	+	+	+	+
	33	211	FMS + strengthening	FMS + aerobic	Improve PPT	1	+	+	+	+	+	+
	34	137	FMS + anodal M1, cathoc	FMS + sham	NA	1	+	+	+	+	+	+
	35	182	FMS Active LENS	FMS sham LENS	Improvement on FMS s	1	+	+	+	+	+	+
	36	222	FMS+ Oestradiol	FMS +Placebo	Change in perceived pa	1	+	+	+	+	+	+
	37	80	FMS+ Acumpucture + tric	FMS+ triciclic + exercise	Improvement pain and	1	+	+	+	+	+	+
	38	198	FMS + Hyperbaric oxigen	FMS + placebo (normal air	NA	1	+	+	+	+	+	+
	39	158	FM +morphine, lidocaine	FM+ placebo	Reduction in pain inter	1	!	!	+	!	!	!

+	Low risk
!	Some concerns
-	High risk
D1	Randomisation process
D2	Deviations from the intended interventions
D3	Missing outcome data
D4	Measurement of the outcome
D5	Selection of the reported result

A summary of the risk of bias assessment for all 39 included randomized controlled trials using the RoB 2 tool, indicating the overall judgments for each domain across studies.

Supplementary S4: Rob2 - Individual quality of RCT studies included (N=39)

**Unique ID**	1	**Study ID**	232	**Assessor**	
**Ref. or Label**	Beradi, 2024	**Aim**	Assignment to intervention (the 'intention-to- treat' effect)		
**Experimental**	FMS + active TENS (Transcutaneous Electrical Nerve	**Comparator**	FMS + placebo TENS / no TENS / HC	**Source**	
**Outcome**	Increase PPT	**Results**		**Weight**	1

**Domain**	**Signalling question**	**Response**	**Comments**
**Bias arising from the randomization process**	1.1 Was the allocation sequence random?	Y	
	1.2 Was the allocation sequence concealed until participants were enrolled and assigned to interventions?	Y	
	1.3 Did the baseline differences between the intervention groups suggest a problem with the randomization process?	N	
	**Risk of bias judgement**	**Low**	
**Bias due to deviations from intended interventions**	2.1.Were the participants aware of their assigned intervention during the trial?	N	
	2.2.Were the carers and people delivering the interventions aware of participants' assigned intervention during the trial?	N	
	2.3. If Y/PY/NI to 2.1 or 2.2: Were there deviations from the intended intervention that arose because of the experimental context?	NA	
	2.4 If Y/PY to 2.3: Were these deviations likely to have affected the outcome?	NA	
	2.5. If Y/PY/NI to 2.4: Were these deviations from intended intervention balanced between groups?	NA	
	2.6 Was an appropriate analysis used to estimate the effect of assignment to intervention?	NI	
	2.7 If N/PN/NI to 2.6: Was there potential for a substantial impact (on the result) of the failure to analyze the participants in the group to which they were randomized?	N	
	**Risk of bias judgement**	**Low**	
**Bias due to missing outcome**	3.1 Were data for this outcome available for all, or nearly all, participants randomized?	Y	
	3.2 If N/PN/NI to 3.1: Is there evidence that the result was not biased by missing outcome data?	NA	
	3.3 If N/PN to 3.2: Could missingness in the outcome depend on its true value?	NA	
	3.4 If Y/PY/NI to 3.3: Is it likely that the	NA	

**data**	missingness in the outcome depended on its true value?		
	**Risk of bias judgement**		
**Bias in measurement of the outcome**	4.1 Was the method of measuring the outcome inappropriate?	N	
	4.2 Could measurement or ascertainment of the outcome have differed between the intervention groups?	N	
	4.3 Were outcome assessors aware of the intervention received by study participants?	NA	
	4.4 If Y/PY/NI to 4.3: Could assessment of the outcome have been influenced by knowledge of intervention received?	NA	Low
	4.5 If Y/PY/NI to 4.4: Is it likely that the assessment of the outcome was influenced by knowledge of intervention received?		
	**Risk of bias judgement**	**NI**	
**Bias in selection of the reported result**	5.1 Were the data that produced this result analyzed in accordance with a pre-specified analysis plan that was finalized before unblinded outcome data were available for analysis?	N	
	5.2 ... multiple eligible outcome measurements (e.g., scales, definitions, time points) within the outcome domain?	Some concerns	Low
	5.3 ... multiple eligible analyses of the data?		
	**Risk of bias judgement**	**Low**	Low
**Overall bias**	**Risk of bias judgement**		

**Unique ID**	2	**Study ID**	41	**Assessor**	
**Ref. or Label**	Boussi-Gross, 2024	**Aim**	Assignment to intervention (the 'intention-to- treat' effect)		
**Experimental**	FMS +Hyperbaric	**Comparator**	FMS + Medication	**Source**	Journal article(s)
**Outcome**	Increase neuroplasticity	**Results**		**Weight**	1
**Domain**	**Signalling question**			**Response**	**Comments**
**Bias arising from the randomization process**	1.1 Was the allocation sequence random?			Y	
	1.2 Was the allocation sequence concealed until participants were enrolled and assigned to interventions?			Y	
	1.3 Did the baseline differences between the intervention groups suggest a problem with the randomization process?			N	
	**Risk of bias judgement**			**Low**	
	2.1.Were the participants aware of their assigned			PY	

**Bias due to deviations from intended interventions**	intervention during the trial?		
	2.2.Were the carers and people delivering the interventions aware of participants' assigned intervention during the trial?	PY	
	2.3. If Y/PY/NI to 2.1 or 2.2: Were there deviations from the intended intervention that arose because of the experimental context?	PY	
	2.4 If Y/PY to 2.3: Were these deviations likely to have affected the outcome?	NI	
	2.5. If Y/PY/NI to 2.4: Were these deviations from intended intervention balanced between groups?	NI	
	2.6 Was an appropriate analysis used to estimate the effect of assignment to intervention?	Y	
	2.7 If N/PN/NI to 2.6: Was there potential for a substantial impact (on the result) of the failure to analyze the participants in the group to which they were randomized?	NA	
	**Risk of bias judgement**	**Some concerns**	
**Bias due to missing outcome data**	3.1 Were data for this outcome available for all, or nearly all, participants randomized?	Y	
	3.2 If N/PN/NI to 3.1: Is there evidence that the result was not biased by missing outcome data?	NA	
	3.3 If N/PN to 3.2: Could missingness in the outcome depend on its true value?	NA	
	3.4 If Y/PY/NI to 3.3: Is it likely that the missingness in the outcome depended on its true value?	NA	
	**Risk of bias judgement**		
**Bias in measurement of the outcome**	4.1 Was the method of measuring the outcome inappropriate?		N
	4.2 Could measurement or ascertainment of the outcome have differed between the intervention groups?		PN
	4.3 Were outcome assessors aware of the intervention received by study participants?		NA
	4.4 If Y/PY/NI to 4.3: Could assessment of the outcome have been influenced by knowledge of intervention received?		NA
	4.5 If Y/PY/NI to 4.4: Is it likely that the assessment of the outcome was influenced by knowledge of intervention received?	Low	
	**Risk of bias judgement**	**NI**	
**Bias in selection of the**	5.1 Were the data that produced this result analyzed in accordance with a pre-specified analysis plan that was finalized before unblinded outcome data were available for analysis?	N	
	5.2 ... multiple eligible outcome measurements (e.g., scales, definitions, time points) within the outcome domain?	N	Low

**reported Result**	5.3 ... multiple eligible analyses of the data?	Some concerns	Low
	**Risk of bias judgement**	**Low**	Low
**Overall bias**	**Risk of bias judgement**		

**Unique ID**	3	**Study ID**	195	**Assessor**	
**Ref. or Label**	Couepel, 2024	**Aim**	Assignment to intervention (the 'intention-to- treat' effect)		
**Experimental**	FMS + educational activity	**Comparator**	FMS + control	**Source**	
**Outcome**	Increase PPT	**Results**		**Weight**	1

**Domain**	**Signalling question**	**Response**	**Comments**
**Bias arising from the randomization process**	1.1 Was the allocation sequence random?	Y	
	1.2 Was the allocation sequence concealed until participants were enrolled and assigned to interventions?	Y	
	1.3 Did the baseline differences between the intervention groups suggest a problem with the randomization process?	N	
	**Risk of bias judgement**	**Low**	
**Bias due to deviations from**	2.1.Were the participants aware of their assigned intervention during the trial?	PN	
	2.2.Were the carers and people delivering the interventions aware of participants' assigned intervention during the trial?	PY	
	2.3. If Y/PY/NI to 2.1 or 2.2: Were there deviations from the intended intervention that arose because of the experimental context?	Y	
	2.4 If Y/PY to 2.3: Were these deviations likely to have affected the outcome?	N	

**intended interventions**	2.5. If Y/PY/NI to 2.4: Were these deviations from intended intervention balanced between groups?	NA	
	2.6 Was an appropriate analysis used to estimate the effect of assignment to intervention?	Y	
	2.7 If N/PN/NI to 2.6: Was there potential for a substantial impact (on the result) of the failure to analyze the participants in the group to which they were randomized?	NA	
	**Risk of bias judgement**	**Some concerns**	
**Bias due to missing outcome data**	3.1 Were data for this outcome available for all, or nearly all, participants randomized?	Y	
	3.2 If N/PN/NI to 3.1: Is there evidence that the result was not biased by missing outcome data?	NA	
	3.3 If N/PN to 3.2: Could missingness in the outcome depend on its true value?	NA	
	3.4 If Y/PY/NI to 3.3: Is it likely that the missingness in the outcome depended on its true value?	NA	
	**Risk of bias judgement**		
**Bias in measurement of the outcome**	4.1 Was the method of measuring the outcome inappropriate?		N
	4.2 Could measurement or ascertainment of the outcome have differed between the intervention groups?		N
	4.3 Were outcome assessors aware of the intervention received by study participants?		NA
	4.4 If Y/PY/NI to 4.3: Could assessment of the outcome have been influenced by knowledge of intervention received?		NA
	4.5 If Y/PY/NI to 4.4: Is it likely that the assessment of the outcome was influenced by knowledge of intervention received?	Low	
	**Risk of bias judgement**		
**Bias in selection of the reported result**	5.1 Were the data that produced this result analyzed in accordance with a pre-specified analysis plan that was finalized before unblinded outcome data were available for analysis?	N	
	5.2 ... multiple eligible outcome measurements (e.g., scales, definitions, time points) within the outcome domain?	N	
	5.3 ... multiple eligible analyses of the data?	Some concerns	Low
	**Risk of bias judgement**		
**Overall bias**	**Risk of bias judgement**		

**Unique ID**	4	**Study ID**	45	**Assessor**	
**Ref. or Label**	Gungormus, 2024	**Aim**	Assignment to intervention (the 'intention-to- treat' effect)		
**Experimental**	FMS +multisensory stim.	**Comparator**	FMS + placebo	**Source**	Journal article(s)
**Outcome**	pain response	**Results**		**Weight**	1
**Domain**	**Signalling question**			**Respon**	**Comments**

		se	
**Bias arising from the randomization process**	1.1 Was the allocation sequence random?	Y	
	1.2 Was the allocation sequence concealed until participants were enrolled and assigned to interventions?	Y	
	1.3 Did the baseline differences between the intervention groups suggest a problem with the randomization process?	PN	
	**Risk of bias judgement**	**Low**	
**Bias due to deviations from intended interventions**	2.1.Were the participants aware of their assigned intervention during the trial?	N	
	2.2.Were the carers and people delivering the interventions aware of participants' assigned intervention during the trial?	N	
	2.3. If Y/PY/NI to 2.1 or 2.2: Were there deviations from the intended intervention that arose because of the experimental context?	NA	
	2.4 If Y/PY to 2.3: Were these deviations likely to have affected the outcome?	NA	
	2.5. If Y/PY/NI to 2.4: Were these deviations from intended intervention balanced between groups?	NA	
	2.6 Was an appropriate analysis used to estimate the effect of assignment to intervention?	Y	
	2.7 If N/PN/NI to 2.6: Was there potential for a substantial impact (on the result) of the failure to analyze the participants in the group to which they were randomized?	NA	
	**Risk of bias judgement**	**Low**	
**Bias due to missing outcome data**	3.1 Were data for this outcome available for all, or nearly all, participants randomized?	Y	
	3.2 If N/PN/NI to 3.1: Is there evidence that the result was not biased by missing outcome data?	NA	
	3.3 If N/PN to 3.2: Could missingness in the outcome depend on its true value?	NA	
	3.4 If Y/PY/NI to 3.3: Is it likely that the missingness in the outcome depended on its true value?	NA	
	**Risk of bias judgement**		
**Bias in measurement of the outcome**	4.1 Was the method of measuring the outcome inappropriate?	PN	
	4.2 Could measurement or ascertainment of the outcome have differed between the intervention groups?	N	
	4.3 Were outcome assessors aware of the intervention received by study participants?	NA	
	4.4 If Y/PY/NI to 4.3: Could assessment of the outcome have been influenced by knowledge of intervention received?	NA	Low
	4.5 If Y/PY/NI to 4.4: Is it likely that the assessment of the outcome was influenced by knowledge of intervention received?		
	**Risk of bias judgement**	**NI**	
**Bias in selection**	5.1 Were the data that produced this result analyzed in accordance with a pre-specified analysis plan that was finalized before unblinded outcome data were available for analysis?	N	
	5.2 ... multiple eligible outcome measurements (e.g., scales, definitions, time points) within the outcome	Some concerns	Low

of the reported result	domain?		
	5.3 ... multiple eligible analyses of the data?		
	**Risk of bias judgement**	**Low**	Low
**Overall bias**	**Risk of bias judgement**		

**Unique ID**	5	**Study ID**	40	**Assessor**	
**Ref. or Label**	Neira, 2024	**Aim**	Assignment to intervention (the 'intention-to- treat' effect)		
**Experimental**	FMS + aquatic therapy (AT)	**Comparator**	FMS + Land-based therapy (LBT)	**Source**	
**Outcome**	Improvement in pain	**Results**		**Weight**	1

**Domain**	**Signalling question**	**Response**	**Comments**
**Bias arising from the randomization process**	1.1 Was the allocation sequence random?	Y	
	1.2 Was the allocation sequence concealed until participants were enrolled and assigned to interventions?	Y	
	1.3 Did the baseline differences between the intervention groups suggest a problem with the randomization process?	N	
	**Risk of bias judgement**	**Low**	
**Bias due to deviations from intended interventions**	2.1.Were the participants aware of their assigned intervention during the trial?	Y	
	2.2.Were the carers and people delivering the interventions aware of participants' assigned intervention during the trial?	Y	
	2.3. If Y/PY/NI to 2.1 or 2.2: Were there deviations from the intended intervention that arose because of the experimental context?	N	
	2.4 If Y/PY to 2.3: Were these deviations likely to have affected the outcome?	NA	
	2.5. If Y/PY/NI to 2.4: Were these deviations from intended intervention balanced between groups?	NA	
	2.6 Was an appropriate analysis used to estimate the effect of assignment to intervention?	Y	
	2.7 If N/PN/NI to 2.6: Was there potential for a substantial impact (on the result) of the failure to analyze the participants in the group to which they were randomized?	NA	
	**Risk of bias judgement**	**Low**	
**Bias due to missing outcome data**	3.1 Were data for this outcome available for all, or nearly all, participants randomized?	Y	
	3.2 If N/PN/NI to 3.1: Is there evidence that the result was not biased by missing outcome data?	NA	
	3.3 If N/PN to 3.2: Could missingness in the outcome depend on its true value?	NA	
	3.4 If Y/PY/NI to 3.3: Is it likely that the missingness in the outcome depended on its true value?	NA	
	**Risk of bias judgement**		
	4.1 Was the method of measuring the outcome inappropriate?		N

**Bias in measurement of the outcome**	4.2 Could measurement or ascertainment of the outcome have differed between the intervention groups?		N
	4.3 Were outcome assessors aware of the intervention received by study participants?		NA
	4.4 If Y/PY/NI to 4.3: Could assessment of the outcome have been influenced by knowledge of intervention received?		NA

	4.5 If Y/PY/NI to 4.4: Is it likely that the assessment of the outcome was influenced by knowledge of intervention received?	Low	
	**Risk of bias judgement**		
**Bias in selection of the reported result**	5.1 Were the data that produced this result analyzed in accordance with a pre-specified analysis plan that was finalized before unblinded outcome data were available for analysis?	N	
	5.2 ... multiple eligible outcome measurements (e.g., scales, definitions, time points) within the outcome domain?	N	
	5.3 ... multiple eligible analyses of the data?	Some concerns	Low
	**Risk of bias judgement**		
**Overall bias**	**Risk of bias judgement**		

**Unique ID**	6	**Study ID**	12	**Assessor**	
**Ref. or Label**	Sanzo, 2024	**Aim**	Assignment to intervention (the 'intention-to- treat' effect)		
**Experimental**	FMS +radial shockwave therapy - RSWT	**Comparator**	FMS + Placebo RSWT	**Source**	
**Outcome**	Improvement PPT	**Results**		**Weight**	1

**Domain**	**Signalling question**	**Response**	**Comments**
**Bias arising from the randomization process**	1.1 Was the allocation sequence random?	Y	
	1.2 Was the allocation sequence concealed until participants were enrolled and assigned to interventions?	Y	
	1.3 Did the baseline differences between the intervention groups suggest a problem with the randomization process?	N	
	**Risk of bias judgement**	**Low**	
**Bias due to deviations from intended interventions**	2.1.Were the participants aware of their assigned intervention during the trial?	N	
	2.2.Were the carers and people delivering the interventions aware of participants' assigned intervention during the trial?	N	
	2.3. If Y/PY/NI to 2.1 or 2.2: Were there deviations from the intended intervention that arose because of the experimental context?	NA	
	2.4 If Y/PY to 2.3: Were these deviations likely to have affected the outcome?	NA	
	2.5. If Y/PY/NI to 2.4: Were these deviations from intended intervention balanced between groups?	NA	
	2.6 Was an appropriate analysis used to estimate the effect of assignment to intervention?	Y	
	2.7 If N/PN/NI to 2.6: Was there potential for a substantial impact (on the result) of the failure to analyze the participants in the group to which they were randomized?	NA	
	**Risk of bias judgement**	**Low**	

**Bias due to missing outcome data**	3.1 Were data for this outcome available for all, or nearly all, participants randomized?	Y	
	3.2 If N/PN/NI to 3.1: Is there evidence that the result was not biased by missing outcome data?	NA	
	3.3 If N/PN to 3.2: Could missingness in the outcome depend on its true value?	NA	
	3.4 If Y/PY/NI to 3.3: Is it likely that the missingness in the outcome depended on its true value?	NA	
	**Risk of bias judgement**		
**Bias in measurement of the outcome**	4.1 Was the method of measuring the outcome inappropriate?		N
	4.2 Could measurement or ascertainment of the outcome have differed between the intervention groups?		N
	4.3 Were outcome assessors aware of the intervention received by study participants?		NA
	4.4 If Y/PY/NI to 4.3: Could assessment of the outcome have been influenced by knowledge of intervention received?		NA
	4.5 If Y/PY/NI to 4.4: Is it likely that the assessment of the outcome was influenced by knowledge of intervention received?	Low	
	**Risk of bias judgement**		
**Bias in selection of the reported result**	5.1 Were the data that produced this result analyzed in accordance with a pre-specified analysis plan that was finalized before unblinded outcome data were available for analysis?	N	
	5.2 ... multiple eligible outcome measurements (e.g., scales, definitions, time points) within the outcome domain?	N	
	5.3 ... multiple eligible analyses of the data?	Some concerns	Low
	**Risk of bias judgement**		
**Overall bias**	**Risk of bias judgement**		

**Unique ID**	7	**Study ID**	231	**Assessor**	
**Ref. or Label**	Ablin, 2023	**Aim**	Assignment to intervention (the 'intention-to- treat' effect)		
**Experimental**	FMS + HBOT-Hyperbaric oxygen therapy	**Comparator**	FMS +medications	**Source**	Journal article(s)
**Outcome**	Improvement of FMS symptoms	**Results**		**Weight**	1

**Domain**	**Signalling question**	**Response**	**Comments**
**Bias arising from the randomization**	1.1 Was the allocation sequence random?	Y	
	1.2 Was the allocation sequence concealed until participants were enrolled and assigned to interventions?	Y	
	1.3 Did the baseline differences between the intervention groups suggest a problem with the randomization process?	N	

**process**	**Risk of bias judgement**	**Low**	
**Bias due to deviations from intended interventions**	2.1.Were the participants aware of their assigned intervention during the trial?	N	
	2.2.Were the carers and people delivering the interventions aware of participants' assigned intervention during the trial?	N	
	2.3. If Y/PY/NI to 2.1 or 2.2: Were there deviations from the intended intervention that arose because of the experimental context?	NA	
	2.4 If Y/PY to 2.3: Were these deviations likely to have affected the outcome?	NA	
	2.5. If Y/PY/NI to 2.4: Were these deviations from intended intervention balanced between groups?	NA	
	2.6 Was an appropriate analysis used to estimate the effect of assignment to intervention?	Y	
	2.7 If N/PN/NI to 2.6: Was there potential for a substantial impact (on the result) of the failure to analyze the participants in the group to which they were randomized?	NA	
	**Risk of bias judgement**	**Low**	
**Bias due to missing outcome data**	3.1 Were data for this outcome available for all, or nearly all, participants randomized?	Y	
	3.2 If N/PN/NI to 3.1: Is there evidence that the result was not biased by missing outcome data?	NA	
	3.3 If N/PN to 3.2: Could missingness in the outcome depend on its true value?	NA	
	3.4 If Y/PY/NI to 3.3: Is it likely that the missingness in the outcome depended on its true value?	NA	
	**Risk of bias judgement**		
**Bias in measurement of the outcome**	4.1 Was the method of measuring the outcome inappropriate?	N	
	4.2 Could measurement or ascertainment of the outcome have differed between the intervention groups?	N	
	4.3 Were outcome assessors aware of the intervention received by study participants?	NA	
	4.4 If Y/PY/NI to 4.3: Could assessment of the outcome have been influenced by knowledge of intervention received?	NA	Low
	4.5 If Y/PY/NI to 4.4: Is it likely that the assessment of the outcome was influenced by knowledge of intervention received?		
	**Risk of bias judgement**	**PY**	
**Bias in selection of the reported result**	5.1 Were the data that produced this result analyzed in accordance with a pre-specified analysis plan that was finalized before unblinded outcome data were available for analysis?	N	
	5.2 ... multiple eligible outcome measurements (e.g., scales, definitions, time points) within the outcome domain?	Low	Low
	5.3 ... multiple eligible analyses of the data?		
	**Risk of bias judgement**	**Low**	Low
**Overall bias**	**Risk of bias judgement**		

**Unique ID**	8	**Study ID**	32	**Assessor**	
**Ref. or Label**	Alsouhibani, 2022	**Aim**	Assignment to intervention (the 'intention-to- treat' effect)		
**Experimental**	FMS + isometric exercise	**Comparator**	HC + isometric exercise	**Source**	Non-commercial trial registry record (e.g., Clin
**Outcome**	Improvement in CPM	**Results**		**Weight**	1

icalTrials.gov record)

Domain	Signalling question	Response	Comments
**Bias arising from the randomization process**	1.1 Was the allocation sequence random?	Y	
	1.2 Was the allocation sequence concealed until participants were enrolled and assigned to interventions?	Y	
	1.3 Did the baseline differences between the intervention groups suggest a problem with the randomization process?	N	
	**Risk of bias judgement**	**Low**	
**Bias due to deviations from intended interventions**	2.1.Were the participants aware of their assigned intervention during the trial?	N	
	2.2.Were the carers and people delivering the interventions aware of participants' assigned intervention during the trial?	Y	
	2.3. If Y/PY/NI to 2.1 or 2.2: Were there deviations from the intended intervention that arose because of the experimental context?	N	
	2.4 If Y/PY to 2.3: Were these deviations likely to have affected the outcome?	NA	
	2.5. If Y/PY/NI to 2.4: Were these deviations from intended intervention balanced between groups?	NA	
	2.6 Was an appropriate analysis used to estimate the effect of assignment to intervention?	NI	
	2.7 If N/PN/NI to 2.6: Was there potential for a substantial impact (on the result) of the failure to analyze the participants in the group to which they were randomized?	N	
	**Risk of bias judgement**	**Low**	
**Bias due to missing outcome data**	3.1 Were data for this outcome available for all, or nearly all, participants randomized?	Y	
	3.2 If N/PN/NI to 3.1: Is there evidence that the result was not biased by missing outcome data?	NA	
	3.3 If N/PN to 3.2: Could missingness in the outcome depend on its true value?	NA	
	3.4 If Y/PY/NI to 3.3: Is it likely that the missingness in the outcome depended on its true value?	NA	
	**Risk of bias judgement**		
**Bias in measurement of the outcome**	4.1 Was the method of measuring the outcome inappropriate?	N	
	4.2 Could measurement or ascertainment of the outcome have differed between the intervention groups?	NI	
	4.3 Were outcome assessors aware of the intervention received by study participants?	PN	
	4.4 If Y/PY/NI to 4.3: Could assessment of the outcome have been influenced by knowledge of intervention received?	NA	Low
	4.5 If Y/PY/NI to 4.4: Is it likely that the assessment of the outcome was influenced by knowledge of intervention received?		
	**Risk of bias judgement**	**NI**	
**Bias in selection**	5.1 Were the data that produced this result analyzed in accordance with a pre-specified analysis plan that was finalized before unblinded outcome data were available for analysis?	N	
	5.2 ... multiple eligible outcome measurements (e.g., scales, definitions, time points) within the outcome	Some concerns	Low

**of the reported result**	domain?		
	5.3 ... multiple eligible analyses of the data?		
	**Risk of bias judgement**	**Low**	Low
**Overall bias**	**Risk of bias judgement**		

**Unique ID**	9	**Study ID**	34	**Assessor**	
**Ref. or Label**	Jaminson, 2022	**Aim**	Assignment to intervention (the 'intention-to- treat' effect)		
**Experimental**	FMS + Transcutaneous nerve stim	**Comparator**	FMS + sham	**Source**	
**Outcome**	Pain reduction	**Results**		**Weight**	1

**Domain**	**Signalling question**	**Response**	**Comments**
**Bias arising from the randomization process**	1.1 Was the allocation sequence random?	Y	
	1.2 Was the allocation sequence concealed until participants were enrolled and assigned to interventions?	Y	
	1.3 Did the baseline differences between the intervention groups suggest a problem with the randomization process?	N	
	**Risk of bias judgement**	**Low**	
**Bias due to deviations from intended interventions**	2.1.Were the participants aware of their assigned intervention during the trial?	N	
	2.2.Were the carers and people delivering the interventions aware of participants' assigned intervention during the trial?	N	
	2.3. If Y/PY/NI to 2.1 or 2.2: Were there deviations from the intended intervention that arose because of the experimental context?	NA	
	2.4 If Y/PY to 2.3: Were these deviations likely to have affected the outcome?	NA	
	2.5. If Y/PY/NI to 2.4: Were these deviations from intended intervention balanced between groups?	NA	
	2.6 Was an appropriate analysis used to estimate the effect of assignment to intervention?	Y	
	2.7 If N/PN/NI to 2.6: Was there potential for a substantial impact (on the result) of the failure to analyze the participants in the group to which they were randomized?	NA	
	**Risk of bias judgement**	**Low**	
**Bias due to missing outcome data**	3.1 Were data for this outcome available for all, or nearly all, participants randomized?	Y	
	3.2 If N/PN/NI to 3.1: Is there evidence that the result was not biased by missing outcome data?	NA	
	3.3 If N/PN to 3.2: Could missingness in the outcome depend on its true value?	NA	
	3.4 If Y/PY/NI to 3.3: Is it likely that the missingness in the outcome depended on its true value?	NA	
	**Risk of bias judgement**		
	4.1 Was the method of measuring the outcome inappropriate?	N	
	4.2 Could measurement or ascertainment of the outcome have differed between the intervention groups?	N	

**Bias in measurement of the outcome**	4.3 Were outcome assessors aware of the intervention received by study participants?	NA	
	4.4 If Y/PY/NI to 4.3: Could assessment of the outcome have been influenced by knowledge of intervention received?	NA	Low
	4.5 If Y/PY/NI to 4.4: Is it likely that the assessment of the outcome was influenced by knowledge of intervention received?		
	**Risk of bias judgement**	**Y**	
**Bias in selection of the reported result**	5.1 Were the data that produced this result analyzed in accordance with a pre-specified analysis plan that was finalized before unblinded outcome data were available for analysis?	N	
	5.2 ... multiple eligible outcome measurements (e.g., scales, definitions, time points) within the outcome domain?	Low	Low
	5.3 ... multiple eligible analyses of the data?		
	**Risk of bias judgement**	**Low**	Low
**Overall bias**	**Risk of bias judgement**		

**Unique ID**	10	**Study ID**	206	**Assessor**	
**Ref. or Label**	Lin, 2022	**Aim**	Assignment to intervention (the 'intention-to- treat' effect)		
**Experimental**	FM + Transcranial alternating current stimulation (HD-tACS)	**Comparator**	FMS + Sham HD-tACS	**Source**	
**Outcome**		**Results**		**Weight**	1

**Domain**	**Signalling question**	**Response**	**Comments**
**Bias arising from the randomization process**	1.1 Was the allocation sequence random?	Y	
	1.2 Was the allocation sequence concealed until participants were enrolled and assigned to interventions?	Y	
	1.3 Did the baseline differences between the intervention groups suggest a problem with the randomization process?	N	
	**Risk of bias judgement**	**Low**	
**Bias due to deviations from intended interventions**	2.1.Were the participants aware of their assigned intervention during the trial?	N	
	2.2.Were the carers and people delivering the interventions aware of participants' assigned intervention during the trial?	N	
	2.3. If Y/PY/NI to 2.1 or 2.2: Were there deviations from the intended intervention that arose because of the experimental context?	NA	
	2.4 If Y/PY to 2.3: Were these deviations likely to have affected the outcome?	NA	
	2.5. If Y/PY/NI to 2.4: Were these deviations from intended intervention balanced between groups?	NA	
	2.6 Was an appropriate analysis used to estimate the effect of assignment to intervention?	Y	
	2.7 If N/PN/NI to 2.6: Was there potential for a substantial impact (on the result) of the failure to analyze	NA	

	the participants in the group to which they were randomized?		
	**Risk of bias judgement**	**Low**	

**Bias due to missing outcome data**	3.1 Were data for this outcome available for all, or nearly all, participants randomized?	Y	
	3.2 If N/PN/NI to 3.1: Is there evidence that the result was not biased by missing outcome data?	NA	
	3.3 If N/PN to 3.2: Could missingness in the outcome depend on its true value?	NA	
	3.4 If Y/PY/NI to 3.3: Is it likely that the missingness in the outcome depended on its true value?	NA	
	**Risk of bias judgement**		
**Bias in measurement of the outcome**	4.1 Was the method of measuring the outcome inappropriate?		N
	4.2 Could measurement or ascertainment of the outcome have differed between the intervention groups?		N
	4.3 Were outcome assessors aware of the intervention received by study participants?		NA
	4.4 If Y/PY/NI to 4.3: Could assessment of the outcome have been influenced by knowledge of intervention received?		NA
	4.5 If Y/PY/NI to 4.4: Is it likely that the assessment of the outcome was influenced by knowledge of intervention received?	Low	
	**Risk of bias judgement**		
**Bias in selection of the reported result**	5.1 Were the data that produced this result analyzed in accordance with a pre-specified analysis plan that was finalized before unblinded outcome data were available for analysis?	N	
	5.2 ... multiple eligible outcome measurements (e.g., scales, definitions, time points) within the outcome domain?	N	
	5.3 ... multiple eligible analyses of the data?	Low	Low
	**Risk of bias judgement**		
**Overall bias**	**Risk of bias judgement**		

**Unique ID**	11	**Study ID**	58	**Assessor**	
**Ref. or Label**	Samartin-Veiga, 2022	**Aim**	Assignment to intervention (the 'intention-to- treat' effect)		
**Experimental**	FMS + TMS (M1, DLPF, OIC)	**Comparator**	FMS + TMS Sham	**Source**	
**Outcome**	Pain intensity	**Results**		**Weight**	1

**Domain**	**Signalling question**	**Response**	**Comments**
**Bias arising from the randomization process**	1.1 Was the allocation sequence random?	Y	
	1.2 Was the allocation sequence concealed until participants were enrolled and assigned to interventions?	Y	
	1.3 Did the baseline differences between the intervention groups suggest a problem with the randomization process?	N	
	**Risk of bias judgement**	**Low**	
	2.1.Were the participants aware of their assigned intervention during the trial?	N	
	2.2.Were the carers and people delivering the interventions aware of participants' assigned intervention during the trial?	N	

**Bias due to deviations from intended interventions**	2.3. If Y/PY/NI to 2.1 or 2.2: Were there deviations from the intended intervention that arose because of the experimental context?	NA	
	2.4 If Y/PY to 2.3: Were these deviations likely to have affected the outcome?	NA	
	2.5. If Y/PY/NI to 2.4: Were these deviations from intended intervention balanced between groups?	NA	
	2.6 Was an appropriate analysis used to estimate the effect of assignment to intervention?	Y	
	2.7 If N/PN/NI to 2.6: Was there potential for a substantial impact (on the result) of the failure to analyze the participants in the group to which they were randomized?	NA	
	**Risk of bias judgement**	**Low**	
**Bias due to missing outcome data**	3.1 Were data for this outcome available for all, or nearly all, participants randomized?	Y	
	3.2 If N/PN/NI to 3.1: Is there evidence that the result was not biased by missing outcome data?	NA	
	3.3 If N/PN to 3.2: Could missingness in the outcome depend on its true value?	NA	
	3.4 If Y/PY/NI to 3.3: Is it likely that the missingness in the outcome depended on its true value?	NA	
	**Risk of bias judgement**		
**Bias in measurement of the outcome**	4.1 Was the method of measuring the outcome inappropriate?		N
	4.2 Could measurement or ascertainment of the outcome have differed between the intervention groups?		N
	4.3 Were outcome assessors aware of the intervention received by study participants?		NA
	4.4 If Y/PY/NI to 4.3: Could assessment of the outcome have been influenced by knowledge of intervention received?		NA
	4.5 If Y/PY/NI to 4.4: Is it likely that the assessment of the outcome was influenced by knowledge of intervention received?	Low	
	**Risk of bias judgement**		
**Bias in selection of the reported result**	5.1 Were the data that produced this result analyzed in accordance with a pre-specified analysis plan that was finalized before unblinded outcome data were available for analysis?	N	
	5.2 ... multiple eligible outcome measurements (e.g., scales, definitions, time points) within the outcome domain?	N	
	5.3 ... multiple eligible analyses of the data?	Some concerns	Low
	**Risk of bias judgement**		
**Overall bias**	**Risk of bias judgement**		

**Unique ID**	12	**Study ID**	64	**Assessor**	
**Ref. or Label**	Izquierdo-Alventosa, 2021	**Aim**	Assignment to intervention (the 'intention-to- treat' effect)		
**Experimental**	FMS + Physical exercise	**Comparator**	FMS + TMS /Control group	**Source**	
**Outcome**	Improvement in pain	**Results**		**Weight**	1
**Domain**	**Signalling question**			**Respons**	**Comments**

		e	
**Bias arising from the randomization process**	1.1 Was the allocation sequence random?	Y	Participants were randomly allocated to 2 different interven-<br><br>tions and a control group.
	1.2 Was the allocation sequence concealed until participants were enrolled and assigned to interventions?	Y	
	1.3 Did the baseline differences between the intervention groups suggest a problem with the randomization process?	N	
	**Risk of bias judgement**	**Low**	
**Bias due to deviations from intended interventions**	2.1.Were the participants aware of their assigned intervention during the trial?	N	
	2.2.Were the carers and people delivering the interventions aware of participants' assigned intervention during the trial?	PN	
	2.3. If Y/PY/NI to 2.1 or 2.2: Were there deviations from the intended intervention that arose because of the experimental context?	NA	
	2.4 If Y/PY to 2.3: Were these deviations likely to have affected the outcome?	NA	
	2.5. If Y/PY/NI to 2.4: Were these deviations from intended intervention balanced between groups?	NA	
	2.6 Was an appropriate analysis used to estimate the effect of assignment to intervention?	Y	
	2.7 If N/PN/NI to 2.6: Was there potential for a substantial impact (on the result) of the failure to analyze the participants in the group to which they were randomized?	NA	
	**Risk of bias judgement**	**Low**	
**Bias due to missing outcome data**	3.1 Were data for this outcome available for all, or nearly all, participants randomized?	Y	
	3.2 If N/PN/NI to 3.1: Is there evidence that the result was not biased by missing outcome data?	NA	
	3.3 If N/PN to 3.2: Could missingness in the outcome depend on its true value?	NA	
	3.4 If Y/PY/NI to 3.3: Is it likely that the missingness in the outcome depended on its true value?	NA	
	**Risk of bias judgement**		
**Bias in measurement of the outcome**	4.1 Was the method of measuring the outcome inappropriate?		N
	4.2 Could measurement or ascertainment of the outcome have differed between the intervention groups?		N
	4.3 Were outcome assessors aware of the intervention received by study participants?		NA
	4.4 If Y/PY/NI to 4.3: Could assessment of the outcome have been influenced by knowledge of intervention received?		NA
	4.5 If Y/PY/NI to 4.4: Is it likely that the assessment of the outcome was influenced by knowledge of intervention received?	Low	
	**Risk of bias judgement**		
**Bias in selection of**	5.1 Were the data that produced this result analyzed in accordance with a pre-specified analysis plan that was finalized before unblinded outcome data were available for analysis?	N	

	5.2 ... multiple eligible outcome measurements (e.g., scales, definitions, time points) within the outcome domain?	N	

the reported result	5.3 ... multiple eligible analyses of the data?	Low	Low
	**Risk of bias judgement**		
**Overall bias**	**Risk of bias judgement**		

**Unique ID**	13	**Study ID**	67	**Assessor**	
**Ref. or Label**	Jaminson, 2021	**Aim**	Assignment to intervention (the 'intention-to- treat' effect)		
**Experimental**	FMS active wearable transcutaneous electrical nerve stimulation	**Comparator**	FMS sham wearable transcutaneous electrical nerve stimulation	**Source**	Journal article(s)
**Outcome**		**Results**		**Weight**	1

**Domain**	**Signalling question**	**Response**	**Comments**
**Bias arising from the randomization process**	1.1 Was the allocation sequence random?	Y	
	1.2 Was the allocation sequence concealed until participants were enrolled and assigned to interventions?	Y	
	1.3 Did the baseline differences between the intervention groups suggest a problem with the randomization process?	N	
	**Risk of bias judgement**	**Low**	
**Bias due to deviations from intended interventions**	2.1.Were the participants aware of their assigned intervention during the trial?	N	
	2.2.Were the carers and people delivering the interventions aware of participants' assigned intervention during the trial?	N	
	2.3. If Y/PY/NI to 2.1 or 2.2: Were there deviations from the intended intervention that arose because of the experimental context?	NA	
	2.4 If Y/PY to 2.3: Were these deviations likely to have affected the outcome?	NA	
	2.5. If Y/PY/NI to 2.4: Were these deviations from intended intervention balanced between groups?	NA	
	2.6 Was an appropriate analysis used to estimate the effect of assignment to intervention?	Y	
	2.7 If N/PN/NI to 2.6: Was there potential for a substantial impact (on the result) of the failure to analyze the participants in the group to which they were randomized?	NA	
	**Risk of bias judgement**	**Low**	
**Bias due to missing outcome data**	3.1 Were data for this outcome available for all, or nearly all, participants randomized?	Y	
	3.2 If N/PN/NI to 3.1: Is there evidence that the result was not biased by missing outcome data?	NA	
	3.3 If N/PN to 3.2: Could missingness in the outcome depend on its true value?	NA	
	3.4 If Y/PY/NI to 3.3: Is it likely that the missingness in the outcome depended on its true value?	NA	
	**Risk of bias judgement**		
	4.1 Was the method of measuring the outcome inappropriate?	N	

**Bias in measurement of the outcome**	4.2 Could measurement or ascertainment of the outcome have differed between the intervention groups?	N	
	4.3 Were outcome assessors aware of the intervention received by study participants?	NA	
	4.4 If Y/PY/NI to 4.3: Could assessment of the outcome have been influenced by knowledge of intervention received?	NA	Low
	4.5 If Y/PY/NI to 4.4: Is it likely that the assessment of the outcome was influenced by knowledge of intervention received?		
	**Risk of bias judgement**	**NI**	
**Bias in selection of the reported result**	5.1 Were the data that produced this result analyzed in accordance with a pre-specified analysis plan that was finalized before unblinded outcome data were available for analysis?	N	
	5.2 ... multiple eligible outcome measurements (e.g., scales, definitions, time points) within the outcome domain?	Some concerns	Low
	5.3 ... multiple eligible analyses of the data?		
	**Risk of bias judgement**	**Low**	Low
**Overall bias**	**Risk of bias judgement**		

**Unique ID**	14	**Study ID**	63	**Assessor**	
**Ref. or Label**	Karamanlioglu, 2021	**Aim**	Assignment to intervention (the 'intention-to- treat' effect)		
**Experimental**	FMS + pregabalin and exercise	**Comparator**	FMS + exercise	**Source**	
**Outcome**		**Results**		**Weight**	1

**Domain**	**Signalling question**	**Response**	**Comments**
**Bias arising from the randomization process**	1.1 Was the allocation sequence random?	Y	
	1.2 Was the allocation sequence concealed until participants were enrolled and assigned to interventions?	Y	
	1.3 Did the baseline differences between the intervention groups suggest a problem with the randomization process?	N	
	**Risk of bias judgement**	**Low**	
**Bias due to deviations from intended interventions**	2.1.Were the participants aware of their assigned intervention during the trial?	N	
	2.2.Were the carers and people delivering the interventions aware of participants' assigned intervention during the trial?	N	
	2.3. If Y/PY/NI to 2.1 or 2.2: Were there deviations from the intended intervention that arose because of the experimental context?	NA	
	2.4 If Y/PY to 2.3: Were these deviations likely to have affected the outcome?	NA	
	2.5. If Y/PY/NI to 2.4: Were these deviations from intended intervention balanced between groups?	NA	
	2.6 Was an appropriate analysis used to estimate the effect of assignment to intervention?	Y	
	2.7 If N/PN/NI to 2.6: Was there potential for a	NA	

	substantial impact (on the result) of the failure to analyze the participants in the group to which they were randomized?		
	**Risk of bias judgement**	**Low**	
**Bias due to missing outcome data**	3.1 Were data for this outcome available for all, or nearly all, participants randomized?	Y	
	3.2 If N/PN/NI to 3.1: Is there evidence that the result was not biased by missing outcome data?	NA	
	3.3 If N/PN to 3.2: Could missingness in the outcome depend on its true value?	NA	
	3.4 If Y/PY/NI to 3.3: Is it likely that the missingness in the outcome depended on its true value?	NA	
	**Risk of bias judgement**		
**Bias in measurement of the outcome**	4.1 Was the method of measuring the outcome inappropriate?		N
	4.2 Could measurement or ascertainment of the outcome have differed between the intervention groups?		N
	4.3 Were outcome assessors aware of the intervention received by study participants?		NA
	4.4 If Y/PY/NI to 4.3: Could assessment of the outcome have been influenced by knowledge of intervention received?		NA
	4.5 If Y/PY/NI to 4.4: Is it likely that the assessment of the outcome was influenced by knowledge of intervention received?	Low	
	**Risk of bias judgement**		
**Bias in selection of the reported result**	5.1 Were the data that produced this result analyzed in accordance with a pre-specified analysis plan that was finalized before unblinded outcome data were available for analysis?	N	
	5.2 ... multiple eligible outcome measurements (e.g., scales, definitions, time points) within the outcome domain?	N	
	5.3 ... multiple eligible analyses of the data?	Some concerns	Low
	**Risk of bias judgement**		
**Overall bias**	**Risk of bias judgement**		

**Unique ID**	15	**Study ID**	71	**Assessor**	
**Ref. or Label**	Sarmento,2020	**Aim**	Assignment to intervention (the 'intention-to- treat' effect)		
**Experimental**	FM +Qigong	**Comparator**	FM + Sham Qigong	**Source**	
**Outcome**	Increase PPT	**Results**		**Weight**	1

**Domain**	**Signalling question**	**Response**	**Comments**
**Bias arising from the randomization**	1.1 Was the allocation sequence random?	Y	
	1.2 Was the allocation sequence concealed until participants were enrolled and assigned to interventions?	Y	
	1.3 Did the baseline differences between the intervention groups suggest a problem with the randomization process?	N	

**process**			

	**Risk of bias judgement**	**Low**	
**Bias due to deviations from intended interventions**	2.1.Were the participants aware of their assigned intervention during the trial?	N	
	2.2.Were the carers and people delivering the interventions aware of participants' assigned intervention during the trial?	N	
	2.3. If Y/PY/NI to 2.1 or 2.2: Were there deviations from the intended intervention that arose because of the experimental context?	NA	
	2.4 If Y/PY to 2.3: Were these deviations likely to have affected the outcome?	NA	
	2.5. If Y/PY/NI to 2.4: Were these deviations from intended intervention balanced between groups?	NA	
	2.6 Was an appropriate analysis used to estimate the effect of assignment to intervention?	Y	
	2.7 If N/PN/NI to 2.6: Was there potential for a substantial impact (on the result) of the failure to analyze the participants in the group to which they were randomized?	NA	
	**Risk of bias judgement**	**Low**	
**Bias due to missing outcome data**	3.1 Were data for this outcome available for all, or nearly all, participants randomized?	Y	
	3.2 If N/PN/NI to 3.1: Is there evidence that the result was not biased by missing outcome data?	NA	
	3.3 If N/PN to 3.2: Could missingness in the outcome depend on its true value?	NA	
	3.4 If Y/PY/NI to 3.3: Is it likely that the missingness in the outcome depended on its true value?	NA	
	**Risk of bias judgement**		
**Bias in measurement of the outcome**	4.1 Was the method of measuring the outcome inappropriate?		N
	4.2 Could measurement or ascertainment of the outcome have differed between the intervention groups?		N
	4.3 Were outcome assessors aware of the intervention received by study participants?		NA
	4.4 If Y/PY/NI to 4.3: Could assessment of the outcome have been influenced by knowledge of intervention received?		NA
	4.5 If Y/PY/NI to 4.4: Is it likely that the assessment of the outcome was influenced by knowledge of intervention received?	Low	
	**Risk of bias judgement**		
**Bias in selection of the reported result**	5.1 Were the data that produced this result analyzed in accordance with a pre-specified analysis plan that was finalized before unblinded outcome data were available for analysis?	N	
	5.2 ... multiple eligible outcome measurements (e.g., scales, definitions, time points) within the outcome domain?	N	
	5.3 ... multiple eligible analyses of the data?	Some concerns	Low
	**Risk of bias judgement**		
**Overall bias**	**Risk of bias judgement**		

Unique ID	16	Study ID	70	Assessor	
Ref. or Label	Udina-Cortés, 2020	Aim	Assignment to intervention (the 'intention-to- treat' effect)		
Experimental	FMS Neuroadaptive electroestimulation	Comparator	FMS + Sham	Source	Journal article(s)
Outcome	Improvement in pain and quality of life	Results		Weight	1

Domain	Signalling question	Response	Comments
Bias arising from the randomization process	1.1 Was the allocation sequence random?	Y	
	1.2 Was the allocation sequence concealed until participants were enrolled and assigned to interventions?	Y	
	1.3 Did the baseline differences between the intervention groups suggest a problem with the randomization process?	N	
	**Risk of bias judgement**	**Low**	
Bias due to deviations from intended interventions	2.1.Were the participants aware of their assigned intervention during the trial?	N	
	2.2.Were the carers and people delivering the interventions aware of participants' assigned intervention during the trial?	N	
	2.3. If Y/PY/NI to 2.1 or 2.2: Were there deviations from the intended intervention that arose because of the experimental context?	NA	
	2.4 If Y/PY to 2.3: Were these deviations likely to have affected the outcome?	NA	
	2.5. If Y/PY/NI to 2.4: Were these deviations from intended intervention balanced between groups?	NA	
	2.6 Was an appropriate analysis used to estimate the effect of assignment to intervention?	NI	
	2.7 If N/PN/NI to 2.6: Was there potential for a substantial impact (on the result) of the failure to analyze the participants in the group to which they were randomized?	PN	
	**Risk of bias judgement**	**Low**	
Bias due to missing outcome data	3.1 Were data for this outcome available for all, or nearly all, participants randomized?	Y	
	3.2 If N/PN/NI to 3.1: Is there evidence that the result was not biased by missing outcome data?	NA	
	3.3 If N/PN to 3.2: Could missingness in the outcome depend on its true value?	NA	
	3.4 If Y/PY/NI to 3.3: Is it likely that the missingness in the outcome depended on its true value?	NA	
	**Risk of bias judgement**		
Bias in measurement of the outcome	4.1 Was the method of measuring the outcome inappropriate?	N	
	4.2 Could measurement or ascertainment of the outcome have differed between the intervention groups?	PN	
	4.3 Were outcome assessors aware of the intervention received by study participants?	NA	
	4.4 If Y/PY/NI to 4.3: Could assessment of the outcome have been influenced by knowledge of intervention received?	NA	Low
	4.5 If Y/PY/NI to 4.4: Is it likely that the assessment of		

	the outcome was influenced by knowledge of intervention received?		
	**Risk of bias judgement**	**NI**	
**Bias in selection of the reported result**	5.1 Were the data that produced this result analyzed in accordance with a pre-specified analysis plan that was finalized before unblinded outcome data were available for analysis?	N	
	5.2 ... multiple eligible outcome measurements (e.g., scales, definitions, time points) within the outcome domain?	Some concerns	Low
	5.3 ... multiple eligible analyses of the data?		
	**Risk of bias judgement**	**Low**	Low
**Overall bias**	**Risk of bias judgement**		

**Unique ID**	17	**Study ID**	86	**Assessor**	
**Ref. or Label**	Izquierdo-Alventosa, 2020a	**Aim**	Assignment to intervention (the 'intention-to- treat' effect)		
**Experimental**	FMS + low physical exercise	**Comparator**	FMS +control	**Source**	
**Outcome**	Pain perception; PPT	**Results**		**Weight**	1

**Domain**	**Signalling question**	**Response**	**Comments**
**Bias arising from the randomization process**	1.1 Was the allocation sequence random?	Y	
	1.2 Was the allocation sequence concealed until participants were enrolled and assigned to interventions?	Y	
	1.3 Did the baseline differences between the intervention groups suggest a problem with the randomization process?	N	
	**Risk of bias judgement**	**Low**	
**Bias due to deviations from intended interventions**	2.1.Were the participants aware of their assigned intervention during the trial?	N	
	2.2.Were the carers and people delivering the interventions aware of participants' assigned intervention during the trial?	Y	
	2.3. If Y/PY/NI to 2.1 or 2.2: Were there deviations from the intended intervention that arose because of the experimental context?	N	
	2.4 If Y/PY to 2.3: Were these deviations likely to have affected the outcome?	NA	
	2.5. If Y/PY/NI to 2.4: Were these deviations from intended intervention balanced between groups?	NA	
	2.6 Was an appropriate analysis used to estimate the effect of assignment to intervention?	Y	
	2.7 If N/PN/NI to 2.6: Was there	NA	

	potential for a substantial impact (on the result) of the failure to analyze the participants in the group to which they were randomized?		
	**Risk of bias judgement**	**Low**	
**Bias due to missing outcome data**	3.1 Were data for this outcome available for all, or nearly all, participants randomized?	Y	
	3.2 If N/PN/NI to 3.1: Is there evidence that the result was not biased by missing outcome data?	NA	
	3.3 If N/PN to 3.2: Could missingness in the outcome depend on its true value?	NA	
	3.4 If Y/PY/NI to 3.3: Is it likely that the missingness in the outcome depended on its true value?	NA	

	**Risk of bias judgement**		
**Bias in measurement of the outcome**	4.1 Was the method of measuring the outcome inappropriate?		N
	4.2 Could measurement or ascertainment of the outcome have differed between the intervention groups?		PN
	4.3 Were outcome assessors aware of the intervention received by study participants?		NA
	4.4 If Y/PY/NI to 4.3: Could assessment of the outcome have been influenced by knowledge of intervention received?		NA
	4.5 If Y/PY/NI to 4.4: Is it likely that the assessment of the outcome was influenced by knowledge of intervention received?	Low	
	**Risk of bias judgement**		
**Bias in selection of the reported result**	5.1 Were the data that produced this result analyzed in accordance with a pre-specified analysis plan that was finalized before unblinded outcome data were available for analysis?	N	
	5.2 ... multiple eligible outcome measurements (e.g., scales, definitions, time points) within the outcome domain?	N	
	5.3 ... multiple eligible analyses of the data?	Low	Low
	**Risk of bias judgement**		
**Overall bias**	**Risk of bias judgement**		

**Unique ID**	18	**Study ID**	17	**Assessor**	
**Ref. or Label**	Amer-Cuenca, 2019	**Aim**	Assignment to intervention (the 'intention-to- treat' effect)		
**Experimental**	FMS + pain neuroscience education-biomedical education	**Comparator**		**Source**	Journal article(s)
**Outcome**	Improvement in pain severety	**Results**	Control	**Weight**	1
**Domain**	**Signalling question**			**Response**	**Comments**
**Bias arising from the**	1.1 Was the allocation sequence random?			Y	
	1.2 Was the allocation sequence concealed until participants were enrolled and assigned to interventions?			Y	
	1.3 Did the baseline differences between the intervention groups suggest a			N	

**randomization process**	problem with the randomization process?		
	**Risk of bias judgement**	**Low**	
**Bias due to deviations from intended interventions**	2.1.Were the participants aware of their assigned intervention during the trial?	N	
	2.2.Were the carers and people delivering the interventions aware of participants' assigned intervention during the trial?	Y	
	2.3. If Y/PY/NI to 2.1 or 2.2: Were there deviations from the intended intervention that arose because of the experimental context?	N	
	2.4 If Y/PY to 2.3: Were these deviations likely to have affected the outcome?	NA	
	2.5. If Y/PY/NI to 2.4: Were these deviations from intended intervention balanced between groups?	NA	
	2.6 Was an appropriate analysis used to estimate the effect of assignment to intervention?	Y	
	2.7 If N/PN/NI to 2.6: Was there potential for a substantial impact (on the result) of the failure to analyze the participants in the group to which they were randomized?	NA	
	**Risk of bias judgement**	**Low**	
**Bias due to missing outcome data**	3.1 Were data for this outcome available for all, or nearly all, participants randomized?	Y	
	3.2 If N/PN/NI to 3.1: Is there evidence that the result was not biased by missing outcome data?	NA	
	3.3 If N/PN to 3.2: Could missingness in the outcome depend on its true value?	NA	
	3.4 If Y/PY/NI to 3.3: Is it likely that the missingness in the outcome depended on its true value?	NA	
	**Risk of bias judgement**		
**Bias in measurement of the outcome**	4.1 Was the method of measuring the outcome inappropriate?	N	
	4.2 Could measurement or ascertainment of the outcome have differed between the intervention groups?	N	
	4.3 Were outcome assessors aware of the intervention received by study participants?	NA	
	4.4 If Y/PY/NI to 4.3: Could assessment of the outcome have been influenced by knowledge of intervention received?	NA	Low
	4.5 If Y/PY/NI to 4.4: Is it likely that the assessment of the outcome was influenced by knowledge of intervention		

	received?		
	**Risk of bias judgement**	**NI**	
**Bias in selection of the reported result**	5.1 Were the data that produced this result analyzed in accordance with a pre-specified analysis plan that was finalized before unblinded outcome data were available for analysis?	N	
	5.2 ... multiple eligible outcome measurements (e.g., scales, definitions, time points) within the outcome domain?	Some concerns	Low
	5.3 ... multiple eligible analyses of the data?		
	**Risk of bias judgement**	**Low**	Low
**Overall bias**	**Risk of bias judgement**		

**Unique ID**	19	**Study ID**	91	**Assessor**	
**Ref. or Label**	Donk, 2019	**Aim**	Assignment to intervention (the 'intention-to- treat' effect)		
**Experimental**	FMS + Tapaentadol	**Comparator**	FMS + Placebo	**Source**	Journal article(s)
**Outcome**	Increase CPM	**Results**		**Weight**	1

**Domain**	**Signalling question**	**Response**	**Comments**
**Bias arising from the randomization process**	1.1 Was the allocation sequence random?	Y	
	1.2 Was the allocation sequence concealed until participants were enrolled and assigned to interventions?	Y	
	1.3 Did the baseline differences between the intervention groups suggest a problem with the randomization process?	N	
	**Risk of bias judgement**	**Low**	
**Bias due to deviations from intended interve**	2.1.Were the participants aware of their assigned intervention during the trial?	N	
	2.2.Were the carers and people delivering the interventions aware of participants' assigned intervention during the trial?	N	
	2.3. If Y/PY/NI to 2.1 or 2.2: Were there deviations from the intended intervention that arose because of the experimental context?	NA	
	2.4 If Y/PY to 2.3: Were these deviations likely to have affected the outcome?	NA	
	2.5. If Y/PY/NI to 2.4: Were these deviations from intended intervention balanced between groups?	NA	

**ntions**	2.6 Was an appropriate analysis used to estimate the effect of assignment to intervention?	Y	
	2.7 If N/PN/NI to 2.6: Was there potential for a substantial impact (on the result) of the failure to analyze the participants in the group to which they were randomized?	NA	
	**Risk of bias judgement**	**Low**	
**Bias due to missing outcome data**	3.1 Were data for this outcome available for all, or nearly all, participants randomized?	PY	
	3.2 If N/PN/NI to 3.1: Is there evidence that the result was not biased by missing outcome data?	NA	
	3.3 If N/PN to 3.2: Could missingness in the outcome depend on its true value?	NA	
	3.4 If Y/PY/NI to 3.3: Is it likely that the missingness in the outcome depended on its true value?	NA	
	**Risk of bias judgement**		
**Bias in measurement of the outcome**	4.1 Was the method of measuring the outcome inappropriate?	N	
	4.2 Could measurement or ascertainment of the outcome have differed between the intervention groups?	PN	
	4.3 Were outcome assessors aware of the intervention received by study participants?	NA	
	4.4 If Y/PY/NI to 4.3: Could assessment of the outcome have been influenced by knowledge of intervention received?	NA	Low
	4.5 If Y/PY/NI to 4.4: Is it likely that the assessment of the outcome was influenced by knowledge of intervention received?		
	**Risk of bias judgement**	**N**	
**Bias in selection of the reported result**	5.1 Were the data that produced this result analyzed in accordance with a pre-specified analysis plan that was finalized before unblinded outcome data were available for analysis?	N	
	5.2 ... multiple eligible outcome measurements (e.g., scales, definitions, time points) within the outcome domain?	Low	Low
	5.3 ... multiple eligible analyses of the data?		
	**Risk of bias judgement**	**Low**	Low
**Overall bias**	**Risk of bias judgement**		

**Unique**	20	**Study**	49	**Assesso**	

ID		ID		r	
**Ref. or Label**	dePaula, 2018	**Aim**	Assignment to intervention (the 'intention-to- treat' effect)		
**Experimental**	FMS LDN/ or tDCS	**Comparator**	FMS with placebo treament	**Source**	Journal article(s)
**Outcome**	Improvement in functionality	**Results**		**Weight**	1
**Domain**	**Signalling question**			**Response**	**Comments**
**Bias arising from the randomization process**	1.1 Was the allocation sequence random?			Y	authors refeered to a published study for more nformation
	1.2 Was the allocation sequence concealed until participants were enrolled and assigned to interventions?			PY	
	1.3 Did the baseline differences between the intervention groups suggest a problem with the randomization process?			PY	
	**Risk of bias judgement**			**Some concerns**	
**Bias due to deviations from intended interventions**	2.1.Were the participants aware of their assigned intervention during the trial?			PY	this is not described in the article/ just mentioned the main study
	2.2.Were the carers and people delivering the interventions aware of participants' assigned intervention during the trial?			PY	
	2.3. If Y/PY/NI to 2.1 or 2.2: Were there deviations from the intended intervention that arose because of the experimental context?			PN	
	2.4 If Y/PY to 2.3: Were these deviations likely to have affected the outcome?			NA	
	2.5. If Y/PY/NI to 2.4: Were these deviations from intended intervention balanced between groups?			NA	
	2.6 Was an appropriate analysis used to estimate the effect of assignment to intervention?			PY	
	2.7 If N/PN/NI to 2.6: Was there potential for a substantial impact (on the result) of the failure to analyze the participants in the group to which they were randomized?			NA	
	**Risk of bias judgement**			**Some concerns**	
**Bias due to missing outcome data**	3.1 Were data for this outcome available for all, or nearly all, participants randomized?			Y	
	3.2 If N/PN/NI to 3.1: Is there evidence that the result was not biased by missing outcome data?			NA	
	3.3 If N/PN to 3.2: Could missingness in the outcome depend on its true value?			NA	
	3.4 If Y/PY/NI to 3.3: Is it likely that the missingness in the outcome depended on			NA	

	its true value?		
	**Risk of bias judgement**		
**Bias in measurement of the outcome**	4.1 Was the method of measuring the outcome inappropriate?	PN	
	4.2 Could measurement or ascertainment of the outcome have differed between the intervention groups?	PN	
	4.3 Were outcome assessors aware of the intervention received by study participants?	NA	
	4.4 If Y/PY/NI to 4.3: Could assessment of the outcome have been influenced by knowledge of intervention received?	NA	Low
	4.5 If Y/PY/NI to 4.4: Is it likely that the assessment of the outcome was influenced by knowledge of intervention received?		
	**Risk of bias judgement**	**NI**	
**Bias in selection of the reported result**	5.1 Were the data that produced this result analyzed in accordance with a pre-specified analysis plan that was finalized before unblinded outcome data were available for analysis?	N	
	5.2 ... multiple eligible outcome measurements (e.g., scales, definitions, time points) within the outcome domain?	Some concerns	Low
	5.3 ... multiple eligible analyses of the data?		
	**Risk of bias judgement**	**Some concerns**	Some concerns
**Overall bias**	**Risk of bias judgement**		

**Unique ID**	21	**Study ID**	30	**Assessor**	
**Ref. or Label**	Pickering, 2018	**Aim**	Assignment to intervention (the 'intention- to- treat' effect)		
**Experimental**	FMS + milnacipran	**Comparator**	FMS + placebo	**Source**	Company-owned trial registry record (e.g., GS
**Outcome**	Increase CPM	**Results**		**Weight**	1

**Domain**	**Signalling question**	**Response**	**Comments**
	1.1 Was the allocation sequence random?	Y	

**Bias arising from the randomization process**	1.2 Was the allocation sequence concealed until participants were enrolled and assigned to interventions?	NI	
	1.3 Did the baseline differences between the intervention groups suggest a problem with the randomization process?	NI	
	**Risk of bias judgement**	**Some concerns**	
**Bias due to deviations from intended interventions**	2.1.Were the participants aware of their assigned intervention during the trial?	PN	
	2.2.Were the carers and people delivering the interventions aware of participants' assigned intervention during the trial?	PN	
	2.3. If Y/PY/NI to 2.1 or 2.2: Were there deviations from the intended intervention that arose because of the experimental context?	NA	
	2.4 If Y/PY to 2.3: Were these deviations likely to have affected the outcome?	NA	
	2.5. If Y/PY/NI to 2.4: Were these deviations from intended intervention balanced between groups?	NA	
	2.6 Was an appropriate analysis used to estimate the effect of assignment to intervention?	PY	
	2.7 If N/PN/NI to 2.6: Was there potential for a substantial impact (on the result) of the failure to analyze the participants in the group to which they were randomized?	NA	
	**Risk of bias judgement**	**Some concerns**	
**Bias due to missing outcome data**	3.1 Were data for this outcome available for all, or nearly all, participants randomized?	PY	
	3.2 If N/PN/NI to 3.1: Is there evidence that the result was not biased by missing outcome data?	NA	
	3.3 If N/PN to 3.2: Could missingness in the outcome depend on its true value?	NA	
	3.4 If Y/PY/NI to 3.3: Is it likely that the missingness in the outcome depended on its true value?	NA	
	**Risk of bias judgement**		
**Bias in measurement of the outco**	4.1 Was the method of measuring the outcome inappropriate?	PN	
	4.2 Could measurement or ascertainment of the outcome have differed between the intervention groups?	NA	
	4.3 Were outcome assessors aware of the intervention received by study participants?	NA	
	4.4 If Y/PY/NI to 4.3: Could assessment of the outcome have been influenced by	NA	

me	knowledge of intervention received?		High
	4.5 If Y/PY/NI to 4.4: Is it likely that the assessment of the outcome was influenced by knowledge of intervention received?		
	**Risk of bias judgement**	**PY**	
**Bias in selection of the reported result**	5.1 Were the data that produced this result analyzed in accordance with a pre-specified analysis plan that was finalized before unblinded outcome data were available for analysis?	N	
	5.2 ... multiple eligible outcome measurements (e.g., scales, definitions, time points) within the outcome domain?	Low	Some concerns
	5.3 ... multiple eligible analyses of the data?		
	**Risk of bias judgement**	**Some concerns**	Some concerns
**Overall bias**	**Risk of bias judgement**		

K Clinical Study Register record)

**Unique ID**	22	**Study ID**	123	**Assessor**	
**Ref. or Label**	Albers, 2017	**Aim**	Assignment to intervention (the 'intention-to- treat' effect)		
**Experimental**	FMS + general osteopathic treatment (GOT) +FMS +osteopathic intervention	**Comparator**		**Source**	
**Outcome**	Pain intensity	**Results**		**Weight**	1

**Domain**	**Signalling question**	**Response**	**Comments**
**Bias arising from the randomization process**	1.1 Was the allocation sequence random?	Y	
	1.2 Was the allocation sequence concealed until participants were enrolled and assigned to interventions?	Y	
	1.3 Did the baseline differences between the intervention groups suggest a problem with the randomization process?	N	
	**Risk of bias judgement**	**Low**	
	2.1.Were the participants aware of their	PY	

	assigned intervention during the trial?		
	2.2.Were the carers and people delivering the interventions aware of participants' assigned intervention during the trial?	PY	
	2.3. If Y/PY/NI to 2.1 or 2.2: Were there deviations from the intended intervention that arose because of the experimental context?	PN	

**Bias due to deviations from intended interventions**	2.4 If Y/PY to 2.3: Were these deviations likely to have affected the outcome?	NA	
	2.5. If Y/PY/NI to 2.4: Were these deviations from intended intervention balanced between groups?	NA	
	2.6 Was an appropriate analysis used to estimate the effect of assignment to intervention?	Y	
	2.7 If N/PN/NI to 2.6: Was there potential for a substantial impact (on the result) of the failure to analyze the participants in the group to which they were randomized?	NA	
	**Risk of bias judgement**	**Low**	
**Bias due to missing outcome data**	3.1 Were data for this outcome available for all, or nearly all, participants randomized?	Y	
	3.2 If N/PN/NI to 3.1: Is there evidence that the result was not biased by missing outcome data?	NA	
	3.3 If N/PN to 3.2: Could missingness in the outcome depend on its true value?	NA	
	3.4 If Y/PY/NI to 3.3: Is it likely that the missingness in the outcome depended on its true value?	NA	
	**Risk of bias judgement**		
**Bias in measurement of the outcome**	4.1 Was the method of measuring the outcome inappropriate?		N
	4.2 Could measurement or ascertainment of the outcome have differed between the intervention groups?		PY
	4.3 Were outcome assessors aware of the intervention received by study participants?		PN
	4.4 If Y/PY/NI to 4.3: Could assessment of the outcome have been influenced by knowledge of intervention received?		NA
	4.5 If Y/PY/NI to 4.4: Is it likely that the assessment of the outcome was influenced by knowledge of intervention received?	Low	
	**Risk of bias judgement**		
**Bias in selection of the reported result**	5.1 Were the data that produced this result analyzed in accordance with a pre-specified analysis plan that was finalized before unblinded outcome data were available for analysis?	N	
	5.2 ... multiple eligible outcome measurements (e.g., scales, definitions, time points) within the outcome domain?	N	
	5.3 ... multiple eligible analyses of the data?	Some concerns	Low
	**Risk of bias judgement**		
**Overall bias**	**Risk of bias judgement**		

**Unique ID**	23	**Study ID**	104	**Assessor**	
**Ref. or Label**	Andrade, 2017	**Aim**	Assignment to intervention (the 'intention- to- treat' effect)		
**Experimental**	FMS + Exercise	**Comparator**	FMS + control	**Source**	
**Outcome**	Increase in VO2 and PPT	**Results**		**Weight**	1

**Domain**	**Signalling question**	**Response**	**Comments**
**Bias arising from the randomization process**	1.1 Was the allocation sequence random?	Y	
	1.2 Was the allocation sequence concealed until participants were enrolled and assigned to interventions?	Y	
	1.3 Did the baseline differences between the intervention groups suggest a problem with the randomization process?	N	
	**Risk of bias judgement**	**Low**	
**Bias due to deviations from intended interventions**	2.1.Were the participants aware of their assigned intervention during the trial?	Y	
	2.2.Were the carers and people delivering the interventions aware of participants' assigned intervention during the trial?	Y	
	2.3. If Y/PY/NI to 2.1 or 2.2: Were there deviations from the intended intervention that arose because of the experimental context?	PN	
	2.4 If Y/PY to 2.3: Were these deviations likely to have affected the outcome?	NA	
	2.5. If Y/PY/NI to 2.4: Were these deviations from intended intervention balanced between groups?	NA	
	2.6 Was an appropriate analysis used to estimate the effect of assignment to intervention?	Y	
	2.7 If N/PN/NI to 2.6: Was there potential for a substantial impact (on the result) of the failure to analyze the participants in the group to which they were randomized?	NA	
	**Risk of bias judgement**	**Low**	
**Bias due to missing outcome data**	3.1 Were data for this outcome available for all, or nearly all, participants randomized?	Y	
	3.2 If N/PN/NI to 3.1: Is there evidence that the result was not biased by missing outcome data?	NA	
	3.3 If N/PN to 3.2: Could missingness in the outcome depend on its true value?	NA	
	3.4 If Y/PY/NI to 3.3: Is it likely that the missingness in the outcome depended on its true value?	NA	

	**Risk of bias judgement**		
**Bias in measurement of the outcome**	4.1 Was the method of measuring the outcome inappropriate?		N
	4.2 Could measurement or ascertainment of the outcome have differed between the intervention groups?		Y
	4.3 Were outcome assessors aware of the intervention received by study participants?		N
	4.4 If Y/PY/NI to 4.3: Could assessment of the outcome have been influenced by knowledge of intervention received?		NA
	4.5 If Y/PY/NI to 4.4: Is it likely that the assessment of the outcome was influenced by knowledge of intervention received?	Low	
	**Risk of bias judgement**		
**Bias in selection of the reported result**	5.1 Were the data that produced this result analyzed in accordance with a pre-specified analysis plan that was finalized before unblinded outcome data were available for analysis?	PN	
	5.2 ... multiple eligible outcome measurements (e.g., scales, definitions, time points) within the outcome domain?	N	
	5.3 ... multiple eligible analyses of the data?	Some concerns	Low
	**Risk of bias judgement**		
**Overall bias**	**Risk of bias judgement**		

**Unique ID**	24	**Study ID**	95	**Assessor**	
**Ref. or Label**	Baumueller, 2017	**Aim**	Assignment to intervention (the 'intention-to- treat' effect)		
**Experimental**	FMS + Electromyogram (EMG) biofeedback	**Comparator**	FMS + control	**Source**	
**Outcome**	Improvement PPT	**Results**		**Weight**	1

**Domain**	**Signalling question**	**Response**	**Comments**
**Bias arising from the randomization process**	1.1 Was the allocation sequence random?	Y	
	1.2 Was the allocation sequence concealed until participants were enrolled and assigned to interventions?	Y	
	1.3 Did the baseline differences between the intervention groups suggest a problem with the randomization process?	N	
	**Risk of bias judgement**	**Low**	
	2.1.Were the participants aware of their	Y	

Bias due to deviations from intended interventions	assigned intervention during the trial?		
	2.2.Were the carers and people delivering the interventions aware of participants' assigned intervention during the trial?	Y	
	2.3. If Y/PY/NI to 2.1 or 2.2: Were there deviations from the intended intervention that arose because of the experimental context?	Y	
	2.4 If Y/PY to 2.3: Were these deviations likely to have affected the outcome?	PN	
	2.5. If Y/PY/NI to 2.4: Were these deviations from intended intervention balanced between groups?	NA	
	2.6 Was an appropriate analysis used to estimate the effect of assignment to intervention?	Y	
	2.7 If N/PN/NI to 2.6: Was there potential for a substantial impact (on the result) of the failure to analyze the participants in the group to which they were randomized?	NA	
	**Risk of bias judgement**	**Low**	
Bias due to missing outcome data	3.1 Were data for this outcome available for all, or nearly all, participants randomized?	Y	
	3.2 If N/PN/NI to 3.1: Is there evidence that the result was not biased by missing outcome data?	NA	
	3.3 If N/PN to 3.2: Could missingness in the outcome depend on its true value?	NA	
	3.4 If Y/PY/NI to 3.3: Is it likely that the missingness in the outcome depended on its true value?	NA	
	**Risk of bias judgement**		
Bias in measurement of	4.1 Was the method of measuring the outcome inappropriate?		N
	4.2 Could measurement or ascertainment of the outcome have differed between the intervention groups?		Y
	4.3 Were outcome assessors aware of the intervention received by study participants?		N

**the outcome**	4.4 If Y/PY/NI to 4.3: Could assessment of the outcome have been influenced by knowledge of intervention received?		NA
	4.5 If Y/PY/NI to 4.4: Is it likely that the assessment of the outcome was influenced by knowledge of intervention received?	Low	
	**Risk of bias judgement**		
**Bias in selection of the reported result**	5.1 Were the data that produced this result analyzed in accordance with a pre-specified analysis plan that was finalized before unblinded outcome data were available for analysis?	N	
	5.2 ... multiple eligible outcome measurements (e.g., scales, definitions, time points) within the outcome domain?	N	
	5.3 ... multiple eligible analyses of the data?	Some concerns	Low
	**Risk of bias judgement**		
**Overall bias**	**Risk of bias judgement**		

**Unique ID**	25	**Study ID**	132	**Assessor**	
**Ref. or Label**	Saral, 2016	**Aim**	Assignment to intervention (the 'intention-to- treat' effect)		
**Experimental**	FMS + interdisciplinary group	**Comparator**	FMS + control group	**Source**	
**Outcome**	Increase PPT and QoL	**Results**		**Weight**	1

**Domain**	**Signalling question**	**Response**	**Comments**
**Bias arising from the randomization process**	1.1 Was the allocation sequence random?	Y	
	1.2 Was the allocation sequence concealed until participants were enrolled and assigned to interventions?	Y	
	1.3 Did the baseline differences between the intervention groups suggest a problem with the randomization process?	N	
	**Risk of bias judgement**	**Low**	
**Bias due to deviati**	2.1.Were the participants aware of their assigned intervention during the trial?	Y	
	2.2.Were the carers and people delivering the interventions aware of participants' assigned intervention during the trial?	Y	
	2.3. If Y/PY/NI to 2.1 or 2.2: Were there deviations from the intended intervention that arose because of the experimental context?	Y	

**ons from intended interventions**	2.4 If Y/PY to 2.3: Were these deviations likely to have affected the outcome?	PY	
	2.5. If Y/PY/NI to 2.4: Were these deviations from intended intervention balanced between groups?	PY	
	2.6 Was an appropriate analysis used to estimate the effect of assignment to intervention?	Y	
	2.7 If N/PN/NI to 2.6: Was there potential for a substantial impact (on the result) of the failure to analyze the participants in the group to which they were randomized?	NA	
	**Risk of bias judgement**	**Some concerns**	
**Bias due to missing outcome data**	3.1 Were data for this outcome available for all, or nearly all, participants randomized?	Y	
	3.2 If N/PN/NI to 3.1: Is there evidence that the result was not biased by missing outcome data?	NA	
	3.3 If N/PN to 3.2: Could missingness in the outcome depend on its true value?	NA	
	3.4 If Y/PY/NI to 3.3: Is it likely that the missingness in the outcome depended on its true value?	NA	
	**Risk of bias judgement**		
**Bias in measurement of the outcome**	4.1 Was the method of measuring the outcome inappropriate?		N
	4.2 Could measurement or ascertainment of the outcome have differed between the intervention groups?		N
	4.3 Were outcome assessors aware of the intervention received by study participants?		NA
	4.4 If Y/PY/NI to 4.3: Could assessment of the outcome have been influenced by knowledge of intervention received?		NA
	4.5 If Y/PY/NI to 4.4: Is it likely that the assessment of the outcome was influenced by knowledge of intervention received?	Low	
	**Risk of bias judgement**		
**Bias in selection of the reported result**	5.1 Were the data that produced this result analyzed in accordance with a pre-specified analysis plan that was finalized before unblinded outcome data were available for analysis?	N	
	5.2 ... multiple eligible outcome measurements (e.g., scales, definitions, time points) within the outcome domain?	N	
	5.3 ... multiple eligible analyses of the data?	Some concerns	Low
	**Risk of bias judgement**		
**Overall bias**	**Risk of bias judgement**		

**Unique ID**	26	**Study ID**	145	**Assessor**	
**Ref. or Label**	Efrati, 2015	**Aim**	Assignment to intervention (the 'intention- to- treat' effect)		
**Experimental**	FMS + HBOT	**Comparator**	FMS control	**Source**	
**Outcome**	Increase in PPT	**Results**		**Weight**	1

Domain	Signalling question	Response	Comments
**Bias arising from the randomization process**	1.1 Was the allocation sequence random?	Y	
	1.2 Was the allocation sequence concealed until participants were enrolled and assigned to interventions?	Y	
	1.3 Did the baseline differences between the intervention groups suggest a problem with the randomization process?	N	
	**Risk of bias judgement**	**Low**	
**Bias due to deviations from intended interventions**	2.1.Were the participants aware of their assigned intervention during the trial?	Y	
	2.2.Were the carers and people delivering the interventions aware of participants' assigned intervention during the trial?	PY	
	2.3. If Y/PY/NI to 2.1 or 2.2: Were there deviations from the intended intervention that arose because of the experimental context?	Y	
	2.4 If Y/PY to 2.3: Were these deviations likely to have affected the outcome?	PN	
	2.5. If Y/PY/NI to 2.4: Were these deviations from intended intervention balanced between groups?	NA	
	2.6 Was an appropriate analysis used to estimate the effect of assignment to intervention?	Y	
	2.7 If N/PN/NI to 2.6: Was there potential for a substantial impact (on the result) of the failure to analyze the participants in the group to which they were randomized?	NA	
	**Risk of bias judgement**	**Low**	
**Bias due to missing outcome data**	3.1 Were data for this outcome available for all, or nearly all, participants randomized?	Y	
	3.2 If N/PN/NI to 3.1: Is there evidence that the result was not biased by missing outcome data?	NA	
	3.3 If N/PN to 3.2: Could missingness in the outcome depend on its true value?	NA	
	3.4 If Y/PY/NI to 3.3: Is it likely that the missingness in the outcome depended on its true value?	NA	

	**Risk of bias judgement**		
**Bias in measurement of the outcome**	4.1 Was the method of measuring the outcome inappropriate?		N
	4.2 Could measurement or ascertainment of the outcome have differed between the intervention groups?		PY
	4.3 Were outcome assessors aware of the intervention received by study participants?		N
	4.4 If Y/PY/NI to 4.3: Could assessment of the outcome have been influenced by knowledge of intervention received?		NA
	4.5 If Y/PY/NI to 4.4: Is it likely that the assessment of the outcome was influenced by knowledge of intervention received?	Low	
	**Risk of bias judgement**		
**Bias in selection of the reported result**	5.1 Were the data that produced this result analyzed in accordance with a pre-specified analysis plan that was finalized before unblinded outcome data were available for analysis?	N	
	5.2 ... multiple eligible outcome measurements (e.g., scales, definitions, time points) within the outcome domain?	N	
	5.3 ... multiple eligible analyses of the data?	Some concerns	Low
	**Risk of bias judgement**		
**Overall bias**	**Risk of bias judgement**		

**Unique ID**	27	**Study ID**	141	**Assessor**	
**Ref. or Label**	Staud, 2015	**Aim**	Assignment to intervention (the 'intention-to- treat' effect)		
**Experimental**	FMS +Milacipran	**Comparator**	FMS +Placebo	**Source**	Journal article(s)

**Outcome**	Change in clinical pain/hyperagelsia	**Results**		**Weight**	1

**Domain**	**Signalling question**	**Response**	**Comments**
**Bias arising from the randomization process**	1.1 Was the allocation sequence random?	Y	
	1.2 Was the allocation sequence concealed until participants were enrolled and assigned to interventions?	PY	
	1.3 Did the baseline differences between the intervention groups suggest a problem with the randomization process?	NI	
	**Risk of bias judgement**	**Low**	
**Bias due to deviations from intended interventions**	2.1.Were the participants aware of their assigned intervention during the trial?	PN	
	2.2.Were the carers and people delivering the interventions aware of participants' assigned intervention during the trial?	PN	
	2.3. If Y/PY/NI to 2.1 or 2.2: Were there deviations from the intended intervention that arose because of the experimental context?	NA	
	2.4 If Y/PY to 2.3: Were these deviations likely to have affected the outcome?	NA	
	2.5. If Y/PY/NI to 2.4: Were these deviations from intended intervention balanced between groups?	NA	
	2.6 Was an appropriate analysis used to estimate the effect of assignment to intervention?	NI	
	2.7 If N/PN/NI to 2.6: Was there potential for a substantial impact (on the result) of the failure to analyze the participants in the group to which they were randomized?	NI	
	**Risk of bias judgement**	**Some concerns**	
**Bias due to missing outcome data**	3.1 Were data for this outcome available for all, or nearly all, participants randomized?	PY	
	3.2 If N/PN/NI to 3.1: Is there evidence that the result was not biased by missing outcome data?	NA	
	3.3 If N/PN to 3.2: Could missingness in the outcome depend on its true value?	NA	
	3.4 If Y/PY/NI to 3.3: Is it likely that the missingness in the outcome depended on its true value?	NA	
	**Risk of bias judgement**		
	4.1 Was the method of measuring the outcome inappropriate?	PN	
	4.2 Could measurement or ascertainment	NI	

Bias in measurement of the outcome	of the outcome have differed between the intervention groups?		
	4.3 Were outcome assessors aware of the intervention received by study participants?	PN	
	4.4 If Y/PY/NI to 4.3: Could assessment of the outcome have been influenced by knowledge of intervention received?	NA	Low
	4.5 If Y/PY/NI to 4.4: Is it likely that the assessment of the outcome was influenced by knowledge of intervention received?		
	**Risk of bias judgement**	**NI**	
**Bias in selection of the reported result**	5.1 Were the data that produced this result analyzed in accordance with a pre-specified analysis plan that was finalized before unblinded outcome data were available for analysis?	N	
	5.2 ... multiple eligible outcome measurements (e.g., scales, definitions, time points) within the outcome domain?	Some concerns	Low
	5.3 ... multiple eligible analyses of the data?		
	**Risk of bias judgement**	**Some concerns**	Some concerns
**Overall bias**	**Risk of bias judgement**		

**Unique ID**	28	**Study ID**	219	**Assessor**	
**Ref. or Label**	Castro-Sanchez, 2014	**Aim**	Assignment to intervention (the 'intention-to- treat' effect)		
**Experimental**	FMS +manual therapy	**Comparator**	FMS + control	**Source**	
**Outcome**	Improve PPT	**Results**		**Weight**	1
**Domain**	**Signalling question**			**Response**	**Comments**
**Bias arising from the randomization process**	1.1 Was the allocation sequence random?			Y	
	1.2 Was the allocation sequence concealed until participants were enrolled and assigned to interventions?			Y	
	1.3 Did the baseline differences between the intervention groups suggest a problem with the randomization process?			N	
	**Risk of bias judgement**			**Low**	
	2.1.Were the participants aware of their assigned intervention during the trial?			N	
	2.2.Were the carers and people delivering the interventions aware of participants' assigned intervention during the trial?			Y	

**Bias due to deviations from intended interventions**	2.3. If Y/PY/NI to 2.1 or 2.2: Were there deviations from the intended intervention that arose because of the experimental context?	Y	
	2.4 If Y/PY to 2.3: Were these deviations likely to have affected the outcome?	N	
	2.5. If Y/PY/NI to 2.4: Were these deviations from intended intervention balanced between groups?	NA	
	2.6 Was an appropriate analysis used to estimate the effect of assignment to intervention?	Y	
	2.7 If N/PN/NI to 2.6: Was there potential for a substantial impact (on the result) of the failure to analyze the participants in the group to which they were randomized?	NA	
	**Risk of bias judgement**	**Low**	
**Bias due to missing outcome data**	3.1 Were data for this outcome available for all, or nearly all, participants randomized?	Y	
	3.2 If N/PN/NI to 3.1: Is there evidence that the result was not biased by missing outcome data?	NA	
	3.3 If N/PN to 3.2: Could missingness in the outcome depend on its true value?	NA	
	3.4 If Y/PY/NI to 3.3: Is it likely that the missingness in the outcome depended on its true value?	NA	
	**Risk of bias judgement**		
**Bias in measurement of the outcome**	4.1 Was the method of measuring the outcome inappropriate?		N
	4.2 Could measurement or ascertainment of the outcome have differed between the intervention groups?		NI
	4.3 Were outcome assessors aware of the intervention received by study participants?		PN
	4.4 If Y/PY/NI to 4.3: Could assessment of the outcome have been influenced by knowledge of intervention received?		NA
	4.5 If Y/PY/NI to 4.4: Is it likely that the assessment of the outcome was influenced by knowledge of intervention received?	Low	
	**Risk of bias judgement**		
**Bias in selection of the reported result**	5.1 Were the data that produced this result analyzed in accordance with a pre-specified analysis plan that was finalized before unblinded outcome data were available for analysis?	N	
	5.2 ... multiple eligible outcome measurements (e.g., scales, definitions, time points) within the outcome domain?	N	
	5.3 ... multiple eligible analyses of the data?	Some concerns	Low
	**Risk of bias judgement**		

**Overall bias**	**Risk of bias judgement**		

**Unique ID**	29	**Study ID**	223	**Assessor**	
**Ref. or Label**	Staud, 2014	**Aim**	Assignment to intervention (the 'intention-to- treat' effect)		
**Experimental**	FMS + 100mg/200mg lidocaine	**Comparator**	FMS + placebo	**Source**	
**Outcome**	Reduce in clinical pain	**Results**		**Weight**	1

**Domain**	**Signalling question**	**Response**	**Comments**
**Bias arising from the randomization process**	1.1 Was the allocation sequence random?	Y	
	1.2 Was the allocation sequence concealed until participants were enrolled and assigned to interventions?	Y	
	1.3 Did the baseline differences between the intervention groups suggest a problem with the randomization process?	N	
	**Risk of bias judgement**	**Low**	
**Bias due to deviations from intended interventions**	2.1.Were the participants aware of their assigned intervention during the trial?	N	
	2.2.Were the carers and people delivering the interventions aware of participants' assigned intervention during the trial?	N	
	2.3. If Y/PY/NI to 2.1 or 2.2: Were there deviations from the intended intervention that arose because of the experimental context?	NA	
	2.4 If Y/PY to 2.3: Were these deviations likely to have affected the outcome?	NA	
	2.5. If Y/PY/NI to 2.4: Were these deviations from intended intervention balanced between groups?	NA	
	2.6 Was an appropriate analysis used to estimate the effect of assignment to intervention?	Y	
	2.7 If N/PN/NI to 2.6: Was there potential for a substantial impact (on the result) of the failure to analyze the participants in the group to which they were randomized?	NA	

	**Risk of bias judgement**	**Low**	
**Bias due to missing outcome data**	3.1 Were data for this outcome available for all, or nearly all, participants randomized?	Y	
	3.2 If N/PN/NI to 3.1: Is there evidence that the result was not biased by missing outcome data?	NA	
	3.3 If N/PN to 3.2: Could missingness in the outcome depend on its true value?	NA	
	3.4 If Y/PY/NI to 3.3: Is it likely that the missingness in the outcome depended on its true value?	NA	
	**Risk of bias judgement**		
**Bias in measurement of the outcome**	4.1 Was the method of measuring the outcome inappropriate?		N
	4.2 Could measurement or ascertainment of the outcome have differed between the intervention groups?		PN
	4.3 Were the outcome assessors aware of the intervention received by study participants?		NA
	4.4 If Y/PY/NI to 4.3: Could assessment of the outcome have been influenced by knowledge of intervention received?		NA
	4.5 If Y/PY/NI to 4.4: Is it likely that the assessment of the outcome was influenced by knowledge of intervention received?	Low	
	**Risk of bias judgement**		
**Bias in selection of the reported result**	5.1 Were the data that produced this result analyzed in accordance with a pre-specified analysis plan that was finalized before unblinded outcome data were available for analysis?	N	
	5.2 ... multiple eligible outcome measurements (e.g., scales, definitions, time points) within the outcome domain?	N	
	5.3 ... multiple eligible analyses of the data?	Some concerns	Low
	**Risk of bias judgement**		
**Overall bias**	**Risk of bias judgement**		

**Unique ID**	30	**Study ID**	117	**Assessor**	
**Ref. or Label**	Casanueva, 2013	**Aim**	Assignment to intervention (the 'intention-to- treat' effect)		
**Experimental**	FMS + dry needling	**Comparator**	FMS + control	**Source**	
**Outcome**	Overall pain score	**Results**		**Weight**	1
**Domain**	**Signalling question**			**Response**	**Comments**

**Bias arising from the randomization process**	1.1 Was the allocation sequence random?	Y	
	1.2 Was the allocation sequence concealed until participants were enrolled and assigned to interventions?	Y	
	1.3 Did the baseline differences between the intervention groups suggest a problem with the randomization process?	N	
	**Risk of bias judgement**	**Low**	
**Bias due to deviations from intended interventions**	2.1.Were the participants aware of their assigned intervention during the trial?	PY	
	2.2.Were the carers and people delivering the interventions aware of participants' assigned intervention during the trial?	N	
	2.3. If Y/PY/NI to 2.1 or 2.2: Were there deviations from the intended intervention that arose because of the experimental context?	Y	
	2.4 If Y/PY to 2.3: Were these deviations likely to have affected the outcome?	N	
	2.5. If Y/PY/NI to 2.4: Were these deviations from intended intervention balanced between groups?	NA	
	2.6 Was an appropriate analysis used to estimate the effect of assignment to intervention?	Y	
	2.7 If N/PN/NI to 2.6: Was there potential for a substantial impact (on the result) of the failure to analyze the participants in the group to which they were randomized?	NA	
	**Risk of bias judgement**	**Some concerns**	
**Bias due to missing outcome data**	3.1 Were data for this outcome available for all, or nearly all, participants randomized?	Y	
	3.2 If N/PN/NI to 3.1: Is there evidence that the result was not biased by missing outcome data?	NA	
	3.3 If N/PN to 3.2: Could missingness in the outcome depend on its true value?	NA	
	3.4 If Y/PY/NI to 3.3: Is it likely that the missingness in the outcome depended on its true value?	NA	
	**Risk of bias judgement**		
**Bias in measurement of the outco**	4.1 Was the method of measuring the outcome inappropriate?		N
	4.2 Could measurement or ascertainment of the outcome have differed between the intervention groups?		PY
	4.3 Were the outcome assessors aware of the intervention received by study participants?		N
	4.4 If Y/PY/NI to 4.3: Could assessment of the outcome have been influenced by		

me	knowledge of intervention received?		NA
	4.5 If Y/PY/NI to 4.4: Is it likely that the assessment of the outcome was influenced by knowledge of intervention received?	Low	
	**Risk of bias judgement**		
**Bias in selection of the reported result**	5.1 Were the data that produced this result analyzed in accordance with a pre-specified analysis plan that was finalized before unblinded outcome data were available for analysis?	N	
	5.2 ... multiple eligible outcome measurements (e.g., scales, definitions, time points) within the outcome domain?	N	
	5.3 ... multiple eligible analyses of the data?	Some concerns	Some concerns
	**Risk of bias judgement**		
**Overall bias**	**Risk of bias judgement**		

**Unique ID**	31	**Study ID**	29	**Assessor**	
**Ref. or Label**	Van Oosterwijck, 2013	**Aim**	Assignment to intervention (the 'intention- to- treat' effect)		
**Experimental**	FMS + pain physiology education	**Comparator**	FMS receiving self management education	**Source**	
**Outcome**	Change on endogenous pain inhibition	**Results**		**Weight**	1

**Domain**	**Signalling question**	**Response**	**Comments**
**Bias arising from the randomization process**	1.1 Was the allocation sequence random?	Y	
	1.2 Was the allocation sequence concealed until participants were enrolled and assigned to interventions?	Y	
	1.3 Did the baseline differences between the intervention groups suggest a problem with the randomization process?	N	
	**Risk of bias judgement**	**Low**	
**Bias due to deviations from intende**	2.1.Were the participants aware of their assigned intervention during the trial?	N	
	2.2.Were the carers and people delivering the interventions aware of participants' assigned intervention during the trial?	N	
	2.3. If Y/PY/NI to 2.1 or 2.2: Were there deviations from the intended intervention that arose because of the experimental context?	NA	
	2.4 If Y/PY to 2.3: Were these deviations likely to have affected the outcome?	NA	

**d interventions**	2.5. If Y/PY/NI to 2.4: Were these deviations from intended intervention balanced between groups?	NA	
	2.6 Was an appropriate analysis used to estimate the effect of assignment to intervention?	Y	
	2.7 If N/PN/NI to 2.6: Was there potential for a substantial impact (on the result) of the failure to analyze the participants in the group to which they were randomized?	NA	
	**Risk of bias judgement**	**Low**	
**Bias due to missing outcome data**	3.1 Were data for this outcome available for all, or nearly all, participants randomized?	Y	
	3.2 If N/PN/NI to 3.1: Is there evidence that the result was not biased by missing outcome data?	NA	
	3.3 If N/PN to 3.2: Could missingness in the outcome depend on its true value?	NA	
	3.4 If Y/PY/NI to 3.3: Is it likely that the missingness in the outcome depended on its true value?	NA	
	**Risk of bias judgement**		
**Bias in measurement of the outcome**	4.1 Was the method of measuring the outcome inappropriate?	N	
	4.2 Could measurement or ascertainment of the outcome have differed between the intervention groups?	N	
	4.3 Were the outcome assessors aware of the intervention received by study participants?	NA	
	4.4 If Y/PY/NI to 4.3: Could assessment of the outcome have been influenced by knowledge of intervention received?	NA	Low
	4.5 If Y/PY/NI to 4.4: Is it likely that the assessment of the outcome was influenced by knowledge of intervention received?		Low
	**Risk of bias judgement**	**NI**	
	5.1 Were the data that produced this result analyzed in accordance with a pre-specified analysis plan that was finalized before unblinded outcome data were available for analysis?	N	

Bias in selection of the reported result	5.2 ... multiple eligible outcome measurements (e.g., scales, definitions, time points) within the outcome domain?	Some concerns	Low
	5.3 ... multiple eligible analyses of the data?		
	**Risk of bias judgement**	**Low**	Low
**Overall bias**	**Risk of bias judgement**		

**Unique ID**	32	**Study ID**	171	**Assessor**	
**Ref. or Label**	Hargrove, 2012	**Aim**	Assignment to intervention (the 'intention-to- treat' effect)		
**Experimental**	FMS + tDCS	**Comparator**	FMS + sham	**Source**	
**Outcome**	Increase PPT	**Results**		**Weight**	1
**Domain**	**Signalling question**			**Response**	**Comments**
**Bias arising from the randomization process**	1.1 Was the allocation sequence random?			Y	
	1.2 Was the allocation sequence concealed until participants were enrolled and assigned to interventions?			Y	
	1.3 Did the baseline differences between the intervention groups suggest a problem with the randomization process?			N	
	**Risk of bias judgement**			**Low**	
**Bias due to deviations from intended interventions**	2.1.Were the participants aware of their assigned intervention during the trial?			N	
	2.2.Were the carers and people delivering the interventions aware of participants' assigned intervention during the trial?			N	
	2.3. If Y/PY/NI to 2.1 or 2.2: Were there deviations from the intended intervention that arose because of the experimental context?			NA	
	2.4 If Y/PY to 2.3: Were these deviations likely to have affected the outcome?			NA	
	2.5. If Y/PY/NI to 2.4: Were these deviations from intended intervention balanced between groups?			NA	
	2.6 Was an appropriate analysis used to estimate the effect of assignment to intervention?			Y	
	2.7 If N/PN/NI to 2.6: Was there potential for a substantial impact (on the result) of the failure to analyze the participants in the group to which they were randomized?			NA	

	**Risk of bias judgement**	**Low**	
**Bias due to missing outcome data**	3.1 Were data for this outcome available for all, or nearly all, participants randomized?	Y	
	3.2 If N/PN/NI to 3.1: Is there evidence that the result was not biased by missing outcome data?	NA	
	3.3 If N/PN to 3.2: Could missingness in the outcome depend on its true value?	NA	
	3.4 If Y/PY/NI to 3.3: Is it likely that the missingness in the outcome depended on its true value?	NA	
	**Risk of bias judgement**		
**Bias in measurement of the outcome**	4.1 Was the method of measuring the outcome inappropriate?		N
	4.2 Could measurement or ascertainment of the outcome have differed between the intervention groups?		PN
	4.3 Were the outcome assessors aware of the intervention received by study participants?		NA
	4.4 If Y/PY/NI to 4.3: Could assessment of the outcome have been influenced by knowledge of intervention received?		NA
	4.5 If Y/PY/NI to 4.4: Is it likely that the assessment of the outcome was influenced by knowledge of intervention received?	Low	
	**Risk of bias judgement**		
**Bias in selection of the reported result**	5.1 Were the data that produced this result analyzed in accordance with a pre-specified analysis plan that was finalized before unblinded outcome data were available for analysis?	Y	
	5.2 ... multiple eligible outcome measurements (e.g., scales, definitions, time points) within the outcome domain?	N	
	5.3 ... multiple eligible analyses of the data?	High	Low
	**Risk of bias judgement**		
**Overall bias**	**Risk of bias judgement**		

**Unique ID**	33	**Study ID**	211	**Assessor**	
**Ref. or Label**	Hooten, 2012	**Aim**	Assignment to intervention (the 'intention-to- treat' effect)		
**Experimental**	FMS + strengthening	**Comparator**	FMS + aerobic	**Source**	
**Outcome**	Improve PPT	**Results**		**Weight**	1

**Domain**	**Signalling question**	**Response**	**Comments**
	1.1 Was the allocation sequence random?	Y	
	1.2 Was the allocation sequence	Y	

**Bias arising from the randomization process**	concealed until participants were enrolled and assigned to interventions?		
	1.3 Did the baseline differences between the intervention groups suggest a problem with the randomization process?	N	
	**Risk of bias judgement**	**Low**	
**Bias due to deviations from intended interventions**	2.1.Were the participants aware of their assigned intervention during the trial?	Y	
	2.2.Were the carers and people delivering the interventions aware of participants' assigned intervention during the trial?	Y	
	2.3. If Y/PY/NI to 2.1 or 2.2: Were there deviations from the intended intervention that arose because of the experimental context?	Y	
	2.4 If Y/PY to 2.3: Were these deviations likely to have affected the outcome?	N	
	2.5. If Y/PY/NI to 2.4: Were these deviations from intended intervention balanced between groups?	NA	
	2.6 Was an appropriate analysis used to estimate the effect of assignment to intervention?	Y	
	2.7 If N/PN/NI to 2.6: Was there potential for a substantial impact (on the result) of the failure to analyze the participants in the group to which they were randomized?	NA	
	**Risk of bias judgement**	**Low**	
**Bias due to missing outcome data**	3.1 Were data for this outcome available for all, or nearly all, participants randomized?	Y	
	3.2 If N/PN/NI to 3.1: Is there evidence that the result was not biased by missing outcome data?	NA	
	3.3 If N/PN to 3.2: Could missingness in the outcome depend on its true value?	NA	
	3.4 If Y/PY/NI to 3.3: Is it likely that the missingness in the outcome depended on its true value?	NA	
	**Risk of bias judgement**		
**Bias in measurement of the outcome**	4.1 Was the method of measuring the outcome inappropriate?		N
	4.2 Could measurement or ascertainment of the outcome have differed between the intervention groups?		PY
	4.3 Were the outcome assessors aware of the intervention received by study participants?		PN
	4.4 If Y/PY/NI to 4.3: Could assessment of the outcome have been influenced by knowledge of intervention received?		NA
	4.5 If Y/PY/NI to 4.4: Is it likely that the assessment of the outcome was influenced by knowledge of intervention	Low	

	received?		
	**Risk of bias judgement**		
**Bias in selection of the reported result**	5.1 Were the data that produced this result analyzed in accordance with a pre-specified analysis plan that was finalized before unblinded outcome data were available for analysis?	N	
	5.2 ... multiple eligible outcome measurements (e.g., scales, definitions, time points) within the outcome domain?	N	
	5.3 ... multiple eligible analyses of the data?	Some concerns	Low
	**Risk of bias judgement**		
**Overall bias**	**Risk of bias judgement**		

**Unique ID**	34	**Study ID**	137	**Assessor**	
**Ref. or Label**	Mendonca, 2011	**Aim**	Assignment to intervention (the 'intention-to- treat' effect)		
**Experimental**	FMS + anodal M1, cathodal SO	**Comparator**	FMS + sham	**Source**	Company-owned trial registry record (e.g., GS
**Outcome**		**Results**		**Weight**	1
**Domain**	**Signalling question**			**Response**	**Comments**
**Bias arising from the randomization**	1.1 Was the allocation sequence random?			Y	
	1.2 Was the allocation sequence concealed until participants were enrolled and assigned to interventions?			Y	

K Clinical Study Register record)

**process**	1.3 Did the baseline differences between the intervention groups suggest a problem with the randomization process?	N	
	**Risk of bias judgement**	**Low**	
**Bias due to deviations from intended interventions**	2.1.Were the participants aware of their assigned intervention during the trial?	N	
	2.2.Were the carers and people delivering the interventions aware of participants' assigned intervention during the trial?	N	
	2.3. If Y/PY/NI to 2.1 or 2.2: Were there deviations from the intended intervention that arose because of the experimental context?	NA	
	2.4 If Y/PY to 2.3: Were these deviations likely to have affected the outcome?	NA	
	2.5. If Y/PY/NI to 2.4: Were these deviations from intended intervention balanced between groups?	NA	
	2.6 Was an appropriate analysis used to estimate the effect of assignment to intervention?	Y	
	2.7 If N/PN/NI to 2.6: Was there potential for a substantial impact (on the result) of the failure to analyze the participants in the group to which they were randomized?	NA	
	**Risk of bias judgement**	**Low**	
**Bias due to missing outcome data**	3.1 Were data for this outcome available for all, or nearly all, participants randomized?	Y	
	3.2 If N/PN/NI to 3.1: Is there evidence that the result was not biased by missing outcome data?	NA	
	3.3 If N/PN to 3.2: Could missingness in the outcome depend on its true value?	NA	
	3.4 If Y/PY/NI to 3.3: Is it likely that the missingness in the outcome depended on its true value?	NA	
	**Risk of bias judgement**		
**Bias in measurement of the outcome**	4.1 Was the method of measuring the outcome inappropriate?		N
	4.2 Could measurement or ascertainment of the outcome have differed between the intervention groups?		PN
	4.3 Were the outcome assessors aware of the intervention received by study participants?		NA
	4.4 If Y/PY/NI to 4.3: Could assessment of the outcome have been influenced by knowledge of intervention received?		NA
	4.5 If Y/PY/NI to 4.4: Is it likely that the assessment of the outcome was influenced by knowledge of intervention received?	Low	

	**Risk of bias judgement**		
**Bias in selection of the reported result**	5.1 Were the data that produced this result analyzed in accordance with a pre-specified analysis plan that was finalized before unblinded outcome data were available for analysis?	N	
	5.2 ... multiple eligible outcome measurements (e.g., scales, definitions, time points) within the outcome domain?	N	
	5.3 ... multiple eligible analyses of the data?	Some concerns	Low
	**Risk of bias judgement**		
**Overall bias**	**Risk of bias judgement**		

**Unique ID**	35	**Study ID**	182	**Assessor**	
**Ref. or Label**	Nelson, 2010	**Aim**	Assignment to intervention (the 'intention-to- treat' effect)		
**Experimental**	FMS Active LENS	**Comparator**	FMS sham LENS	**Source**	Company-owned trial registry record (e.g., GS
**Outcome**	Improvement on FMS symptoms	**Results**		**Weight**	1

**Domain**	**Signalling question**	**Response**	**Comments**
**Bias arising from the randomization process**	1.1 Was the allocation sequence random?	Y	
	1.2 Was the allocation sequence concealed until participants were enrolled and assigned to interventions?	Y	
	1.3 Did the baseline differences between the intervention groups suggest a problem with the randomization process?	N	
	**Risk of bias judgement**	**Low**	
**Bias due to deviations from intended interventions**	2.1.Were the participants aware of their assigned intervention during the trial?	N	
	2.2.Were the carers and people delivering the interventions aware of participants' assigned intervention during the trial?	N	
	2.3. If Y/PY/NI to 2.1 or 2.2: Were there deviations from the intended intervention that arose because of the experimental context?	NA	
	2.4 If Y/PY to 2.3: Were these deviations likely to have affected the outcome?	NA	
	2.5. If Y/PY/NI to 2.4: Were these deviations from intended intervention balanced between groups?	NA	
	2.6 Was an appropriate analysis used to	Y	

	estimate the effect of assignment to intervention?		
	2.7 If N/PN/NI to 2.6: Was there potential for a substantial impact (on the result) of the failure to analyze the participants in the group to which they were randomized?	NA	
	**Risk of bias judgement**	**Low**	
**Bias due to missing outcome data**	3.1 Were data for this outcome available for all, or nearly all, participants randomized?	Y	
	3.2 If N/PN/NI to 3.1: Is there evidence that the result was not biased by missing outcome data?	NA	
	3.3 If N/PN to 3.2: Could missingness in the outcome depend on its true value?	NA	
	3.4 If Y/PY/NI to 3.3: Is it likely that the missingness in the outcome depended on its true value?	NA	
	**Risk of bias judgement**		
**Bias in measurement of the outcome**	4.1 Was the method of measuring the outcome inappropriate?	N	
	4.2 Could measurement or ascertainment of the outcome have differed between the intervention groups?	N	
	4.3 Were the outcome assessors aware of the intervention received by study participants?	NA	
	4.4 If Y/PY/NI to 4.3: Could assessment of the outcome have been influenced by knowledge of intervention received?	NA	Low
	4.5 If Y/PY/NI to 4.4: Is it likely that the assessment of the outcome was influenced by knowledge of intervention received?		
	**Risk of bias judgement**	**NI**	
**Bias in selection of the reported result**	5.1 Were the data that produced this result analyzed in accordance with a pre-specified analysis plan that was finalized before unblinded outcome data were available for analysis?	N	
	5.2 ... multiple eligible outcome measurements (e.g., scales, definitions, time points) within the outcome domain?	Some concerns	Low
	5.3 ... multiple eligible analyses of the data?		
	**Risk of bias judgement**	**Low**	Low
**Overall bias**	**Risk of bias judgement**		

K Clinical Study Register record); Grant database summary (e.g., NIH RePORTER, Research Councils UK Gateway to Research)

**Unique ID**	36	**Study ID**	222	**Assessor**	

**Ref. or Label**	Stening et al., 2010	**Aim**	Assignment to intervention (the 'intention-to- treat' effect)		
**Experimental**	FMS + Oestradiol	**Comparator**	FMS +Placebo	**Source**	Journal article(s)
**Outcome**	Change in perceived pain	**Results**		**Weight**	1

**Domain**	**Signalling question**	**Response**	**Comments**
**Bias arising from the randomization process**	1.1 Was the allocation sequence random?	Y	
	1.2 Was the allocation sequence concealed until participants were enrolled and assigned to interventions?	PY	
	1.3 Did the baseline differences between the intervention groups suggest a problem with the randomization process?	PN	
	**Risk of bias judgement**	**Low**	
**Bias due to deviations from intended interventions**	2.1.Were the participants aware of their assigned intervention during the trial?	N	
	2.2.Were the carers and people delivering the interventions aware of participants' assigned intervention during the trial?	N	
	2.3. If Y/PY/NI to 2.1 or 2.2: Were there deviations from the intended intervention that arose because of the experimental context?	NA	
	2.4 If Y/PY to 2.3: Were these deviations likely to have affected the outcome?	NA	
	2.5. If Y/PY/NI to 2.4: Were these deviations from intended intervention balanced between groups?	NA	
	2.6 Was an appropriate analysis used to estimate the effect of assignment to intervention?	Y	
	2.7 If N/PN/NI to 2.6: Was there potential for a substantial impact (on the result) of the failure to analyze the participants in the group to which they were randomized?	NA	
	**Risk of bias judgement**	**Low**	
**Bias due to missing outcome data**	3.1 Were data for this outcome available for all, or nearly all, participants randomized?	Y	
	3.2 If N/PN/NI to 3.1: Is there evidence that the result was not biased by missing outcome data?	NA	
	3.3 If N/PN to 3.2: Could missingness in the outcome depend on its true value?	NA	

	3.4 If Y/PY/NI to 3.3: Is it likely that the missingness in the outcome depended on its true value?	NA	
	**Risk of bias judgement**		
**Bias in measurement of the outcome**	4.1 Was the method of measuring the outcome inappropriate?	N	
	4.2 Could measurement or ascertainment of the outcome have differed between the intervention groups?	NI	
	4.3 Were the outcome assessors aware of the intervention received by study participants?	PN	
	4.4 If Y/PY/NI to 4.3: Could assessment of the outcome have been influenced by knowledge of intervention received?	NA	Low
	4.5 If Y/PY/NI to 4.4: Is it likely that the assessment of the outcome was influenced by knowledge of intervention received?		
	**Risk of bias judgement**	**NI**	
**Bias in selection of the reported result**	5.1 Were the data that produced this result analyzed in accordance with a pre-specified analysis plan that was finalized before unblinded outcome data were available for analysis?	N	
	5.2 ... multiple eligible outcome measurements (e.g., scales, definitions, time points) within the outcome domain?	Some concerns	Low
	5.3 ... multiple eligible analyses of the data?		
	**Risk of bias judgement**	**Low**	Low
**Overall bias**	**Risk of bias judgement**		

**Unique ID**	37	**Study ID**	80	**Assessor**	
**Ref. or Label**	Targino, 2008	**Aim**	Assignment to intervention (the 'intention-to- treat' effect)		
**Experimental**	FMS + Acumpucture + triciclic + exercise	**Comparator**	FMS + triciclic + exercise	**Source**	
**Outcome**	Improvement pain and quality of live	**Results**		**Weight**	1

**Domain**	**Signalling question**	**Response**	**Comments**
**Bias arising**	1.1 Was the allocation sequence random?	Y	
	1.2 Was the allocation sequence concealed until participants were enrolled and assigned to interventions?	Y	

**from the randomization process**	1.3 Did the baseline differences between the intervention groups suggest a problem with the randomization process?	N	
	**Risk of bias judgement**	**Low**	
**Bias due to deviations from intended interventions**	2.1.Were the participants aware of their assigned intervention during the trial?	N	
	2.2.Were the carers and people delivering the interventions aware of participants' assigned intervention during the trial?	Y	
	2.3. If Y/PY/NI to 2.1 or 2.2: Were there deviations from the intended intervention that arose because of the experimental context?	N	
	2.4 If Y/PY to 2.3: Were these deviations likely to have affected the outcome?	NA	
	2.5. If Y/PY/NI to 2.4: Were these deviations from intended intervention balanced between groups?	NA	
	2.6 Was an appropriate analysis used to estimate the effect of assignment to intervention?	Y	
	2.7 If N/PN/NI to 2.6: Was there potential for a substantial impact (on the result) of the failure to analyze the participants in the group to which they were randomized?	NA	
	**Risk of bias judgement**	**Low**	
**Bias due to missing outcome data**	3.1 Were data for this outcome available for all, or nearly all, participants randomized?	Y	
	3.2 If N/PN/NI to 3.1: Is there evidence that the result was not biased by missing outcome data?	NA	
	3.3 If N/PN to 3.2: Could missingness in the outcome depend on its true value?	NA	
	3.4 If Y/PY/NI to 3.3: Is it likely that the missingness in the outcome depended on its true value?	NA	
	**Risk of bias judgement**		
**Bias in measurement of the outcome**	4.1 Was the method of measuring the outcome inappropriate?		N
	4.2 Could measurement or ascertainment of the outcome have differed between the intervention groups?		NI
	4.3 Were the outcome assessors aware of the intervention received by study participants?		PN
	4.4 If Y/PY/NI to 4.3: Could assessment of the outcome have been influenced by knowledge of intervention received?		NA
	4.5 If Y/PY/NI to 4.4: Is it likely that the	Low	

	assessment of the outcome was influenced by knowledge of intervention received?		
	**Risk of bias judgement**		
**Bias in selection of the reported result**	5.1 Were the data that produced this result analyzed in accordance with a pre-specified analysis plan that was finalized before unblinded outcome data were available for analysis?	N	
	5.2 ... multiple eligible outcome measurements (e.g., scales, definitions, time points) within the outcome domain?	N	
	5.3 ... multiple eligible analyses of the data?	Some concerns	Low
	**Risk of bias judgement**		
**Overall bias**	**Risk of bias judgement**		

**Unique ID**	38	**Study ID**	198	**Assessor**	
**Ref. or Label**	Yildiz, 2004	**Aim**	Assignment to intervention (the 'intention-to- treat' effect)		
**Experimental**	FMS + Hyperbaric oxygen- HBO	**Comparator**	FMS + placebo (normal air)	**Source**	
**Outcome**		**Results**		**Weight**	1

**Domain**	**Signalling question**	**Response**	**Comments**
**Bias arising from the randomization process**	1.1 Was the allocation sequence random?	Y	
	1.2 Was the allocation sequence concealed until participants were enrolled and assigned to interventions?	Y	
	1.3 Did the baseline differences between the intervention groups suggest a problem with the randomization process?	N	
	**Risk of bias judgement**	**Low**	
**Bias due to deviations from intende**	2.1.Were the participants aware of their assigned intervention during the trial?	N	
	2.2.Were the carers and people delivering the interventions aware of participants' assigned intervention during the trial?	N	
	2.3. If Y/PY/NI to 2.1 or 2.2: Were there deviations from the intended intervention that arose because of the experimental context?	NA	
	2.4 If Y/PY to 2.3: Were these deviations likely to have affected the outcome?	NA	

**d interventions**	2.5. If Y/PY/NI to 2.4: Were these deviations from intended intervention balanced between groups?	NA	
	2.6 Was an appropriate analysis used to estimate the effect of assignment to intervention?	Y	
	2.7 If N/PN/NI to 2.6: Was there potential for a substantial impact (on the result) of the failure to analyze the participants in the group to which they were randomized?	NA	
	**Risk of bias judgement**	**Low**	
**Bias due to missing outcome data**	3.1 Were data for this outcome available for all, or nearly all, participants randomized?	Y	
	3.2 If N/PN/NI to 3.1: Is there evidence that the result was not biased by missing outcome data?	NA	
	3.3 If N/PN to 3.2: Could missingness in the outcome depend on its true value?	NA	
	3.4 If Y/PY/NI to 3.3: Is it likely that the missingness in the outcome depended on its true value?	NA	
	**Risk of bias judgement**		
**Bias in measurement of the outcome**	4.1 Was the method of measuring the outcome inappropriate?		N
	4.2 Could measurement or ascertainment of the outcome have differed between the intervention groups?		N
	4.3 Were the outcome assessors aware of the intervention received by study participants?		NA
	4.4 If Y/PY/NI to 4.3: Could assessment of the outcome have been influenced by knowledge of intervention received?		NA
	4.5 If Y/PY/NI to 4.4: Is it likely that the assessment of the outcome was influenced by knowledge of intervention received?	Low	
	**Risk of bias judgement**		
**Bias in selection of the reported result**	5.1 Were the data that produced this result analyzed in accordance with a pre-specified analysis plan that was finalized before unblinded outcome data were available for analysis?	N	
	5.2 ... multiple eligible outcome measurements (e.g., scales, definitions, time points) within the outcome domain?	N	
	5.3 ... multiple eligible analyses of the data?	Some concerns	Low
	**Risk of bias judgement**		
**Overall bias**	**Risk of bias judgement**		

**Unique ID**	39	**Study ID**	158	**Assessor**	
**Ref. or Label**	Sorensen, 1995	**Aim**	Assignment to intervention (the 'intention- to- treat' effect)		
**Experimental**	FM +morphine, lidocaine, and ketamine	**Comparator**	FM + placebo	**Source**	
**Outcome**	Reduction in pain intensity	**Results**		**Weight**	1

**Domain**	**Signalling question**	**Response**	**Comments**
**Bias arising from the randomization process**	1.1 Was the allocation sequence random?	Y	
	1.2 Was the allocation sequence concealed until participants were enrolled and assigned to interventions?	NI	
	1.3 Did the baseline differences between the intervention groups suggest a problem with the randomization process?	PN	
	**Risk of bias judgement**	**Some concerns**	
**Bias due to deviations from intended interventions**	2.1.Were the participants aware of their assigned intervention during the trial?	N	
	2.2.Were the carers and people delivering the interventions aware of participants' assigned intervention during the trial?	PN	
	2.3. If Y/PY/NI to 2.1 or 2.2: Were there deviations from the intended intervention that arose because of the experimental context?	NA	
	2.4 If Y/PY to 2.3: Were these deviations likely to have affected the outcome?	NA	
	2.5. If Y/PY/NI to 2.4: Were these deviations from intended intervention balanced between groups?	NA	
	2.6 Was an appropriate analysis used to estimate the effect of assignment to intervention?	NI	
	2.7 If N/PN/NI to 2.6: Was there potential for a substantial impact (on the result) of the failure to analyze the participants in the group to which they were randomized?	NI	
	**Risk of bias judgement**	**Some concerns**	
**Bias due to**	3.1 Were data for this outcome available for all, or nearly all, participants randomized?	N	
	3.2 If N/PN/NI to 3.1: Is there evidence that the result was not biased by missing outcome data?	N	

**missing outcome data**	3.3 If N/PN to 3.2: Could missingness in the outcome depend on its true value?	PN	
	3.4 If Y/PY/NI to 3.3: Is it likely that the missingness in the outcome depended on its true value?	NA	
	**Risk of bias judgement**		
**Bias in measurement of the outcome**	4.1 Was the method of measuring the outcome inappropriate?		N
	4.2 Could measurement or ascertainment of the outcome have differed between the intervention groups?		NI
	4.3 Were the outcome assessors aware of the intervention received by study participants?		NI
	4.4 If Y/PY/NI to 4.3: Could assessment of the outcome have been influenced by knowledge of intervention received?		NI
	4.5 If Y/PY/NI to 4.4: Is it likely that the assessment of the outcome was influenced by knowledge of intervention received?	High	
	**Risk of bias judgement**		
**Bias in selection of the reported result**	5.1 Were the data that produced this result analyzed in accordance with a pre-specified analysis plan that was finalized before unblinded outcome data were available for analysis?	N	
	5.2 ... multiple eligible outcome measurements (e.g., scales, definitions, time points) within the outcome domain?	N	
	5.3 ... multiple eligible analyses of the data?	Some concerns	Low
	**Risk of bias judgement**		
**Overall bias**	**Risk of bias judgement**		

Detailed risk of bias assessments for each included study based on RoB 2 domains and signaling questions, including responses and judgments with supporting comments when applicable.

Supplementary S5: STROBE from nonRCT studies included (n=93)

id	author	year	title	doi	Title		Introduction		Methods									Results					Discussion					
					1		2	3	4	5	6	7	8	9	10	11	12	13	14	15	16	17	18	19	20	21	22	Final score
					Title	abstract	rationale	objectives	study design	setting	participants	variables	data sources	bias	study size	antitative variab	tatistical method	participants	descrip. data	outcome	main results	other analyses	Key results	Limitations	Interpretation	Generalizability	Fundings	total
26	Marshall, A and	2024	Small fibre pathology, sma	DOI: 10.1038/s41	0	0	0,5	1	0	1	1	1	0,5	0	0	1	0	1	1	1	1	0	1	1	1	1	1	15
93	Castelo-Branco,	2024	Cross-sectional and longit	DOI: 10.1002/pri.	0	1	0,5	0,5	1	1	1	1	1	0	0	1	1	1	1	1	1	1	1	1	1	1	1	19
42	Aoe, T. and Kaw	2024	Functional connectivity	DOI: 10.1038/s4	1	1	1	1	1	1	1	1	1	0	0	1	1	1	1	1	1	1	1	1	1	1	1	21
44	Gil-Ugidos, A. a	2024	Conditioned pain modulat	DOI: 10.1038/s41	0	1	1	1	0,5	1	1	1	1	0	0	1	1	1	0,5	1	1	1	1	1	1	1	1	19
46	Tapia-Haro, R.M	2023	Serum VEGF and CGRP Bio	DOI: 10.3390/ijms	1	1	1	1	1	1	1	1	1	0	0	1	1	1	1	1	1	1	1	1	1	1	1	21
47	Kumar, A. and Y	2023	Exploring pain status and	DOI: 10.25259/IJP	0	1	1	1	0	0,5	1	1	0,5	0	0	1	1	1	1	1	1	1	1	1	1	1	1	18
53	Cigaran-Mendez	2023	Pain extent is associated	DOI: 10.1080/03	0	0,5	0,5	1	0,5	1	1	1	0,5	0	1	1	1	1	1	1	1	0	1	1	1	0,5	1	17,5
54	Leone et al.	2023	Small-fibre pathology ha	DOI: 10.1093/br	0	0	1	1	1	1	1	1	1	0	0	1	1	1	1	1	1	1	1	1	1	1	1	19
36	Bao, JD and Ros	2023	Interplay between noxic	DOI: 10.3389/fn	0	0,5	0	1	1	1	1	1	1	0	0	1	1	1	1	1	1	1	1	1	1	1	1	18,5
8	Baumler et al	2023	Strong and aversive colc	DOI: 10.1038/s4	0	1	0,5	1	1	1	0,5	1	0,5	0	0	1	0	1	1	1	1	1	1	1	1	1	1	17,5
238	Soldatelli, M. an	2023	Primary somatosensory	DOI:10.3344/kjp	0	1	1	1	1	1	1	1	1	0	1	1	1	1	1	1	1	1	1	1	1	1	1	21
233	Serrano, P.V. and	2022	Association between de	DOI: 10.3389/fn	1	1	1	1	1	1	1	1	1	0	1	1	1	1	1	1	1	1	1	1	1	1	1	22
10	Castelo-Branco,	2022	Temporal Summation in	DOI: 10.3389/fp	0	1	1	1	1	1	1	1	1	0	0	1	1	1	1	1	1	1	1	1	1	1	1	20
15	Berwick, RJ and	2022	Aftersensations and ligering	10.1093/pm/pnac	0	0,5	0,5	1	1	1	1	1	1	0	0	1	1	1	1	1	1	1	1	0,5	1	0,5	1	18
18	Weber et al	2022	Fibromyalgia-associated	DOI: 10.1371/jo	0	0,5	0,5	1	1	1	1	1	1	0	0	1	1	1	1	1	1	1	1	1	1	1	1	19
14	Rehm et al	2021	Pain matters for central	DOI: 10.1097/PR	0	0	1	1	0,5	1	1	1	1	0	0	1	1	1	1	1	1	1	1	1	1	1	1	18,5
16	van Campen, CN	2021	Orthostatic stress testing i	DOI: 10.22541/au	0	0,5	1	1	0,5	1	1	1	0,5	0	0	0,5	0,5	0,5	1	1	1	1	1	1	1	1	1	17
23	Staud et al	2021	Fibromyalgia Patients A	DOI:10.1016/j.jp	0	0	1	1	0	1	1	1	1	0	1	0	1	1	1	1	1	0	1	1	1	1	1	17
20	Pickering et al.	2020	Electrochemical Skin Co	DOI. 10.1111/pa	0	1	0	1	0,5	0,5	1	1	1	0	0	0,5	1	0,5	1	1	0,5	0	0,5	1	0,5	0,5	1	14
2	Gerhardta et al.	2017	Conditioned pain modu	DOI: 10.1097/j.p	0	1	1	1	1	0,5	0,5	1	1	0	0	1	1	1	1	1	1	0	1	1	1	1	1	18
13	Da Silva et al.	2013	Quantitative sensory tes	DOI: 10.1007/s0	0	0	0,5	0,5	0	1	1	1	1	0	0	1	1	1	1	1	1	1	1	1	1	1	1	17
4	Desmeules et al.	2003	Neurophysiologic evider	DOI 10.1002/art	0	0,5	1	1	0	1	1	1	1	1	1	1	1	1	1	1	1	1	1	0,5	0,5	1	1	19,5
5	Hurtig et al.	2001	Quantitative sensory tes	DOI: 10.1097/00	0	1	1	1	1	1	1	1	1	0	0	1	1	1	1	1	1	1	1	1	1	1	1	20
25	Bruijn et al.	2011	Relevance of physical fitne	DOI: 10.1097/RHU	0	0	1	1	0,5	0,5	1	1	1	0	0	1	1	1	1	1	1	1	1	1	1	0,5	1	17,5
28	Smith et al.	2008	Habituation and sensitiz	DOI: 10.1016/j.p	0	0,5	1	0,5	0	0	1	1	1	1	0	1	1	1	1	1	1	1	1	1	1	1	1	18
31	Brietzke et al.	2019	Potency of descending	DOI: 10.1097/M	0	1	1	1	1	1	1	1	1	1	1	1	1	1	1	1	1	1	1	1	1	1	1	22
33	Schoen et al.	2016	A novel paradigm to eva	DOI: 10.2147/JP	0	0,5	0,5	0,5	0	1	1	1	1	0	0	1	1	1	1	1	1	0	1	1	1	1	1	16,5
37	Staud, R and Vie	2001	Abnormal sensitization an	DOI: 10.1016/S03	0	0,5	1	1	1	0,5	0,5	1	0,5	0	0	1	0,5	1	1	1	1	1	1	1	1	1	1	17,5
38	Ernberg, M and	2003	Effects on muscle pain b	DOI: 10.1016/S0	0,5	1	1	1	1	1	1	1	1	0	0	1	1	1	1	1	1	0	1	1	1	0,5	0	18
56	Tour et al.	2022	The OPRM1 gene and in	DOI: 10.1371/jo	0	0,5	1	1	0,5	0,5	1	1	1	0	0	1	1	1	1	1	1	1	1	1	1	1	1	18,5
60	"Franco" deOlive	2022	Hyper-connectivity betv	DOI: 10.1371/jo	0	1	1	1	1	1	1	1	1	0	1	1	1	1	1	1	1	1	1	1	1	1	1	21
61	Fanton, S. and S	2022	The translocator protein	DOI: 10.1097/j.p	0	0,5	1	1	1	1	1	1	1	0	0	1	1	1	1	1	1	0	1	1	1	1	1	18,5
65	Soldatelli et al	2021	Mapping of predictors o	DOI: 10.1177/20	0	1	1	1	1	1	1	1	1	0	1	1	1	1	1	1	1	1	1	1	1	1	1	21
81	Kaziyama et al.	2020	Sifting the wheat from t	DOI: 10.1002/ej	0	1	1	1	1	1	1	0,5	1	0	0	0,5	0,5	1	0,5	0	0,5	0	0,5	0,5	1	0,5	1	14
82	Uygur-Kucukseyi	2020	Decreased neural inhibi	DOI: 10.1016/j.n	1	1	1	1	1	1	1	1	1	0	0	1	1	1	1	1	1	1	1	1	1	1	1	21
83	Han, C.-L. and S	2020	Serum proteome profile	DOI: 10.1038/s4	0	0,5	0,5	0,5	0	1	1	1	0,5	0	0	1	0	1	0,5	1	1	0,5	1	1	1	0,5	1	14,5
72	Falaguera-Vera,	2020	Pressure point thresh	DOI: 10.3390/i	0	0,5	0,5	1	1	1	1	1	1	0	0	1	1	1	1	1	1	1	1	0,5	1	0,5	1	18
90	Evdokimov et al.	2019	Reduction of skin inner	DOI: 10.1002/an	0	0	0	1	1	1	1	1	1	0	0	1	1	1	1	1	1	1	1	1	1	0	1	17
78	Merriwether et	2018	Physical activity is relate	DOI: 10.1186/s1	0	1	1	1	1	1	1	1	1	0	0	1	1	1	1	1	1	1	1	1	1	1	1	20
76	Jespersen, A and	2007	Computerized cuff pres	DOI: 10.1016/j.p	0,5	1	1	1	1	1	1	1	1	0	0	1	1	1	1	1	1	1	1	1	1	1	1	20,5
77	Yuan, SLK and B	2013	EFFECTS OF SHIATSU IN	DOI: 10.1016/j.j	0,5	1	1	1	1	1	1	1	1	0	0	1	1	1	1	1	0,5	0	1	0,5	1	0,5	1	18
79	Kosek et al.	1997	Modulatory influence o	DOI: 10.1016/s0	0	0,5	1	0,5	1	1	1	1	1	0	0	1	1	1	0,5	1	1	0	1	0,5	0,5	0,5	1	16
94	Hassett et al.	2012	Pain is associated with s	DOI: 10.1016/j.j	0	0,5	1	1	1	1	1	1	1	0	0	1	1	1	1	1	1	0,5	1	1	1	1	1	19
96	Montoya, P and	2005	Altered processing of pa	DOI: 10.1016/j.e	0	1	1	1	0,5	1	1	1	1	0	0	1	1	1	1	1	1	1	1	1	1	1	1	19,5
98	de la Coba, P an	2017	Responses to Slowly Re	DOI: 10.1093/pn	0,5	1	1	1	0,5	1	1	1	1	0	0	1	1	1	1	1	1	1	1	1	1	1	1	20
107	Wodehouse et a	2018	A pilot study investigatir	DOI: 10.1177/20	0	0	1	1	0	1	1	1	1	0	0	1	1	1	1	1	1	1	1	1	1	0,5	1	17,5
108	Eken, A. and GV	2018	Association of Fine Motor	DOI: 10.1080/002	0	0,5	1	1	0,5	1	1	1	1	0	0	1	1	1	1	1	1	1	1	1	1	1	1	19
109	Galvez-Sv"nchez	2018	Cognitive deficits in fibr	DOI: 10.1371/jo	0,5	1	1	1	0	1	1	1	1	0	0	1	1	1	1	1	1	1	1	1	1	1	1	19,5
110	De La Coba, P. a	2018	Slowly Repeated Evoked	DOI: 10.1097/PS	0,5	1	1	1	1	1	1	1	1	0	0	1	1	1	1	1	1	1	1	1	1	1	1	20,5
111	Harper et al.	2018	Resting Functional Conn	DOI: 10.1016/j.j	0	1	1	1	1	1	1	1	1	0	0	1	1	1	1	1	1	1	1	1	1	1	1	20
112	Potvin et al.	2016	Pain facilitation and pai	DOI: 10.1097/j.p	0	0,5	0,5	0,5	0	0	1	0	0	0	1	1	0	1	1	1	1	1	1	1	1	1	1	14,5
116	Graven-Nielsen,	2000	Ketamine reduces musc	DOI: 10.1016/S0	0,5	1	1	1	0	1	0,5	1	1	0	0	1	1	1	1	1	1	1	1	1	1	1	1	19
118	Suman, AL and E	2009	One-year efficacy of a 3-	PMID: 19327223	0,5	1	1	1	1	1	1	1	1	0	0	1	1	1	1	1	1	1	1	1	1	1	1	20,5
124	Barbero et al.	2017	Pain extent is associated	DOI 10.1007/s10	0	1	1	0	0	1	1	1	1	0	1	1	1	1	1	1	1	0	1	1	1	1	1	18
125	Freitas, R.P.D.A.	2017	Impacts of social suppor	DOI: 10.1016/j.r	0	1	1	1	1	1	1	1	1	0	0	1	1	1	1	1	1	1	1	0	0,5	0,5	0	17
129	Staud, R and Ro	2003	Ratings of experimental pa	DOI: 10.1016/S03	0	0,5	1	0,5	0,5	0,5	0,5	1	1	0	0	1	1	1	1	1	1	1	1	1	1	1	1	17,5
130	Diers, M and Ko	2008	Pain ratings and somato	DOI: 10.1097/W	0,5	0,5	1	1	1	1	1	1	1	0	0	1	1	1	1	1	0,5	1	1	1	1	1	1	20
133	Forti, M. and Za	2016	Lung function, respirato	DOI: 10.4187/re	0,5	1	1	1	0,5	1	1	1	1	0	0	1	1	1	1	1	1	1	1	1	1	1	1	20
138	Gomez-Perretta,	2016	Evaluation of the accura	PMID: 26812139	1	1	1	1	1	1	1	1	1	0	0	1	1	1	1	1	1	1	1	1	1	1	1	21
140	Qin et al.	2015	Thermal quantitative se	DOI 10.1007/s11	0	0	1	1	0	0,5	0,5	1	1	0	1	1	1	1	1	1	1	1	1	1	1	1	1	18
143	Soriano-Maldon	2015	Association of cardiores	DOI: 10.1007/s0	0	1	0,5	1	0,5	1	1	1	1	0	0	1	1	1	1	1	1	1	1	0,5	1	1	1	18,5
144	Kim, J. and Logg	2015	The somatosensory link	DOI: 10.1002/ar	0	1	1	1	1	1	1	1	1	0	0	1	1	1	1	1	1	1	1	1	1	1	1	20
150	Zamuner, A.R. a	2015	Effects of a hydrotherap	PMID: 25786047	0	1	1	1	0,5	1	1	1	1	0	0	1	1	1	1	1	1	1	1	1	1	1	1	19,5
153	Price, DD and St	2002	Enhanced temporal sur	DOI: 10.1016/S0	0,5	1	1	1	1	1	1	1	1	0	0	1	1	1	1	1	1	1	1	1	1	1	1	20,5
154	Staud, R and Cr	2008	Brain activity associated	DOI: 10.1016/j.e	0,5	1	1	1	1	1	1	1	1	0	0	1	1	1	1	1	1	1	1	1	1	1	1	20,5
158	Sorensen, J and	1995	Pain analysis in patients	DOI: 10.3109/03	0	1	0,5	1	0	0	1	1	0	0	0	0	0	1	1	1	0	1	1	1	1	1	1	13,5
159	Ge, HY and Nie,	2009	Contribution of the loca	1016/j.pain.2009	0	1	1	1	0	0,5	1	1	0	0	0	1	1	1	0	1	1	1	1	1	1	0,5	1	16
163	Staud, R. and W	2014	Slow temporal summati	DOI: 10.1371/jo	0,5	1	1	1	1	1	1	1	1	0	0	1	1	1	1	1	1	0	1	1	1	1	1	19,5
164	Bokarewa, M.I.	2014	Smoking is associated w	DOI: 10.1155/20	0	0,5	0,5	0,5	0	1	1	1	0	0	0	1	1	0	1	1	1	1	1	0,5	1	0,5	1	14,5
165	Belenguer-Priet	2013	Specificity and sensitivit	PMID: 24373366	0,5	1	1	1	1	1	1	1	1	0	0	1	1	1	1	1	1	1	1	1	1	1	1	20,5
166	Paul-Savoie et a	2012	Is the deficit in pain inhi	DOI: 10.2174/18	0	0,5	0,5	1	0	0,5	1	1	1	0	0	1	1	1	1	1	1	1	1	1	1	1	1	17,5
167	Hassett, A.L. an	2012	Pain is associated with t	DOI: 10.1016/j.j	0	1	1	1	0,5	0	1	1	1	0	0	1	1	1	1	1	1	1	1	1	1	1	1	18,5
180	Tastekin, N and	2010	Discriminative Value of	DOI: 10.1111/j.1	0	1	1	1	1	1	1	1	0	0	0	1	1	1	0	1	1	0	1	0	1	0,5	1	15,5
188	Pacheco-Barrios	2023	Motor Cortex Inhibition	DOI: 10.3390/bi	1	1	1	1	1	1	1	1	1	0	0	1	1	1	1	1	1	1	1	1	1	1	1	21
197	Kendall, SA and	2003	Differences in sensory t	DOI: 10.1300/J0	0,5	0,5	0,5	1	0	1	0,5	1	1	0	0	1	0,5	0,5	1	1	1	1	1	1	1	1	1	17
201	Ozgocmen, S. an	2006	Comparison of pain thresh	DOI: 10.1080/136	0,5	1	1	1	1	1	1	1	1	0	0	1	1	1	1	1	1	1	1	1	1	1	1	20,5
208	Ernberg, M. and	1997	Short-term effect of glu	PMID: 9610315	0	1	1	1	1	1	1	1	1	0	0	1	1	1	1	1	1	0	1	0	1	1	1	18
210	Staud, R and Ro	2005	Isometric exercise has o	DOI: 10.1016/j.p	1	1	1	1	0,5	1	1	1	1	0	0	1	0,5	1	1	1	1	0	1	0	1	1	1	18
212	Castro-Sanchez,	2012	Gender Differences in P	DOI: 10.1111/j.1	0,5	1	1	1	0,5	1	1	1	1	0	0	1	1	1	1	1	1	1	1	1	1	1	1	20
214	Ellingson, LD an	2018	Catastrophizing Interfer	DOI: 10.1093/pn	0	1	1	1	0	1	1	1	1	0	0	1	1	1	1	1	1	1	1	1	1	1	1	19
215	Geisser, ME and	2007	The association betwee	DOI: 10.1016/j.e	1	1	1	1	0	0	1	0,5	1	0	0	1	0	0,5	0,5	1	1	0	1	1	1	0,5	1	15
216	Staud, R and Vie	2004	Spatial summation of he	DOI: 10.1016/j.p	1	1	1	1	0,5	1	1	1	1	0	0	1	1	1	1	1	1	1	1	1	1	1	1	20,5
217	Berardi, G and S	2021	Impact of isometric and	DOI: 10.1007/s0	0	0	1	1	1	1	1	1	1	0	0	1	1	1	1	1	1	1	1	1	1	1	1	19
218	Burgmer, M and	2012	Cerebral mechanisms	DOI: 10.1002/j.	0	0,5	1	1	1	1	1	1	1	0	0	1	1	1	1	1	1	1	1	1	1	0,5	1	19
221	Luciano, JV and	2016	Functional Status, Quali	DOI: 10.1097/AJ	0	0,5	0,5	1	0,5	1	0,5	1	1	0	0,5	1	1	1	1	1	1	1	1	0	0,5	0,5	1	16,5
224	Vandenbroucke,	2014	Fibromyalgia patients ar	DOI: 10.3758/s1	1	1	1	1	1	1	1	1	1	0	0	1	1	1	1	1	1	1	1	1	1	1	1	21
229	Staud, R and Pri	2004	Body pain area and pair	DOI: 10.1016/j.j	0	0,5	1	0,5	0	1	0,5	1	1	0	0	1	1	0,5	1	1	1	0	1	0,5	0,5	0	1	14
230	Oudejans	2016	th outcomes of skin biopsi	: 10.1038/srep23	0	0,5	0,5	1	0	1	0,5	1	1	0	0	1	1	1	1	1	1	0	1	0,5	0,5	0,5	0	14
234	Giesecke et al.	2005	The relationship betwee	DOI 10.1002/art	0	0,5	1	1	0	0,5	1	1	1	0	0	0	1	1	1	1	1	1	1	1	1	0,5	1	16,5
235	Uçeyler et al.	2013	Small fibre pathology in	DOI:10.1093/bra	0	1	1	1	0,5	1	1	1	1	0	0	1	1	1	1	1	1	1	1	1	1	1	1	19,5
236	Crettaz et al.	2013	Stress-Induced Allodyni	DOI: 10.1371/jo	0	0	1	0,5	0	0	0	1	1	0	0	1	1	1	1	1	1	1	1	1	0	1	1	14,5

id	author	year	title	doi	Title		Introduction		Methods									Results					Discussion					
					1		2	3	4	5	6	7	8	9	10	11	12	13	14	15	16	17	18	19	20	21	22	Final score
					Title	abstract	rationale	objectives	study design	setting	participants	variables	data sources	bias	study size	antitative variab	tatistical method	participants	descrip. data	outcome	main results	other analyses	Key results	Limitations	Interpretation	Generalizability	Fundings	total
237	Martinez-Jauan	2012	Pain sensitivity in fibron	DOI: 10.1002/j.1	0	1	1	1	1	1	1	1	1	0	0	1	1	1	1	1	1	1	1	1	1	1	1	20
																												0
																												0

Comprehensive table of raw data extracted from each included study on Quantitative Sensory Testing (QST), including technique type (static or dynamic), body site tested, equipment used, methodology, and outcome parameters.
